# Foot-and-mouth disease virus non-structural protein 2B downregulates the RLR signaling pathway *via* degradation of RIG-I and MDA5

**DOI:** 10.3389/fimmu.2022.1020262

**Published:** 2022-09-29

**Authors:** Asela Weerawardhana, Md Bashir Uddin, Joo-Hyung Choi, Prabuddha Pathinayake, Sung Ho Shin, Kiramage Chathuranga, Jong-Hyeon Park, Jong-Soo Lee

**Affiliations:** ^1^ College of Veterinary Medicine, Chungnam National University, Daejeon, Republic of Korea; ^2^ Department of Medicine, Sylhet Agricultural University, Sylhet, Bangladesh; ^3^ Department of Microbiology and Immunology, University of Texas Medical Branch, Galveston, TX, United States; ^4^ Foot and Mouth Disease Division, Animal Quarantine and Inspection Agency, Anyang, South Korea; ^5^ Wildlife Disease Response Team, National Institute of Wildlife Disease Control and Prevention (NIWDC), Gwangju, Republic of Korea; ^6^ Immune Health Program, Hunter Medical Research Institute, University of Newcastle, Newcastle, NSW, Australia

**Keywords:** foot and mouth disease virus (FMDV), 2B, RIG-I, MDA5, RNF125

## Abstract

Foot-and-mouth disease virus (FMDV) is a single-stranded, positive-sense RNA virus containing at least 13 proteins. Many of these proteins show immune modulation capabilities. As a non-structural protein of the FMDV, 2B is involved in the rearrangement of the host cell membranes and the disruption of the host secretory pathway as a viroporin. Previous studies have also shown that FMDV 2B plays a role in the modulation of host type-I interferon (IFN) responses through the inhibition of expression of RIG-I and MDA5, key cytosolic sensors of the type-I IFN signaling. However, the exact molecular mechanism is poorly understood. Here, we demonstrated that FMDV 2B modulates host IFN signal pathway by the degradation of RIG-I and MDA5. FMDV 2B targeted the RIG-I for ubiquitination and proteasomal degradation by recruiting E3 ubiquitin ligase ring finger protein 125 (RNF125) and also targeted MDA5 for apoptosis-induced caspase-3- and caspase-8-dependent degradation. Ultimately, FMDV 2B significantly inhibited RNA virus-induced IFN-β production. Importantly, we identified that the C-terminal amino acids 126-154 of FMDV 2B are essential for 2B-mediated degradation of the RIG-I and MDA5. Collectively, these results provide a clearer understanding of the specific molecular mechanisms used by FMDV 2B to inhibit the IFN responses and a rational approach to virus attenuation for future vaccine development.

## Introduction

The innate immune signaling cascade plays a crucial role in the antiviral immune responses elicited by various viruses, resulting in the induction of type-I interferons (IFN), inflammatory cytokines, and IFN-stimulated genes (ISGs) (1–3). Pattern recognition receptors (PRRs), including toll-like receptors (TLRs), retinoic acid-inducible gene-I (RIG-I)-like receptors (RLRs), and Nucleotide-binding and oligomerization domain (NOD)-like receptors (NLRs), recognize pathogen-associated molecular patterns (PAMPs) (1, 4, 5). Among the PRRs, the significant members of the RLRs family, RIG-I and melanoma differentiation-associated gene 5 (MDA5) play a pivotal role in sensing cytosolic viral RNAs (1, 4, 6, 7), a critical role in the recognition of picornaviruses (1, 8, 9). After sensing the viral RNA in the cytoplasm by RIG-I or MDA5, they interact with the downstream mitochondrial antiviral signaling protein (MAVS) (10–13) through their tandem N-terminal caspase recruitment domains (CARDs) and activate type-I IFN responses and proinflammatory responses *via* downstream molecules such as TANK-binding kinase 1 (TBK1)/Inhibitor of nuclear factor kappa-B kinase subunit epsilon (IKKϵ), Interferon regulatory factor 3 (IRF3), Nuclear factor-kappa B (NF-κB), etc. (14, 15).

RIG-I plays a significant role in maintaining antiviral immunity by preventing the spread of the virus and is involved in host immune homeostasis due to the presence of several regulatory molecules (16). In addition, post-translational modifications (PTMs), including ubiquitination, phosphorylation, and acetylation, are fundamental regulatory mechanisms involved in the activation or the inactivation of RIG-I (17). Also, MDA5 is another major intracellular sensor for viral dsRNA, and several positive and negative regulatory molecules are involved in the activation or the inactivation of MDA5 to maintain the host immune homeostasis after a viral infection (16). Among the various regulatory mechanisms, one of the less-studied facts related to the regulation of MDA5 is the caspase-3- and caspase-8-dependent cleavage of MDA5 (18). These important sensing molecules are a major target for immune evasion of viruses.

Foot-and-mouth disease (FMD) is a highly contagious vesicular disease in cloven-hoofed animals. It is one of the most economically devastating diseases that are considered to be a significant concern in animal health (19, 20). The etiologic agent of FMD is foot-and-mouth disease virus (FMDV), the prototype member of the *Aphthovirus* genus in the *Picornaviridae* family. FMDV has seven known serotypes (A, O, Asia, C, SAT1, SAT2, and SAT3) (21). The FMDV virion consists of a single-stranded, positive-sense RNA genome of about 8.5 kb in length and enclosed by four structural proteins to form an icosahedral capsid (9). The genomic RNA is translated into a single, long open reading frame for a polyprotein, and subsequently, the polyprotein is cleaved by various proteases, resulting in several intermediate or structural proteins (VP1-VP4) and non-structural proteins (L^pro^, 2A, 2B, 2C, 3A, 3B, 3C^pro^ and 3D) (22).

Among FMDV non-structural proteins, 2B is involved in the interaction with the endoplasmic reticulum (ER) membrane through the predicted two hydrophobic domains for the viral replication (23) and is also related to the permeability of the host cell membrane and the disruption of the cellular secretory pathway (24–27). Studies on the 2B proteins of other family members of *Picornaviridae* show different functions of the 2B protein. Poliovirus 2B protein blocks cellular protein secretion (28), and coxsackievirus 2B protein facilitates virus release by modifying membrane permeability (29). Moreover, recent studies have shown that FMDV 2B protein plays the role of a negative regulator of RLR-mediated type-I IFN signaling by targeting RIG-I, LGP2, and MDA5 (23, 27, 30). However, the exact molecular mechanism through which FMDV 2B targets these molecules is not yet clear. In this study, we demonstrated the precise molecular mechanism by which FMDV 2B mediates the degradation of RIG-I and MDA5.

## Results

### FMDV 2B negatively regulates antiviral immune responses

To evade and suppress the host innate immune system, viruses must regulate the type-I IFN signal transduction. Previous studies have shown that FMDV 2B plays an important role in regulating IFN-dependent immune responses (23, 27). However, the molecular mechanisms underlying FMDV 2B-mediated inhibition of the host antiviral innate immune responses remain unclear. To reconfirm that FMDV 2B is involved in the downregulation of IFN-β signaling, we screened FMDV proteins for IFN-β luciferase activity. When human embryonic kidney (HEK293T) cells were co-transfected with the indicated plasmids, we found that FMDV 2B and FMDV 3C significantly inhibited RIG-I-induced IFN-β reporter activity (**Supplementary Figure 1A**). For a specific study on FMDV 2B, we evaluated IFN antagonism mediated by FMDV 2B in different serotypes of FMDV and observed that the 2B proteins of all FMDV serotypes downregulated IFN-β promoter activity in a dose-dependent manner. In particular, compared with other serotypes, SAT1, SAT2, and SAT3 showed noticeable reductions in the luciferase activity (**Supplementary Figure 1B**). These results suggest that FMDV 2B inhibits IFN-β promoter activity.

**Figure 1:**
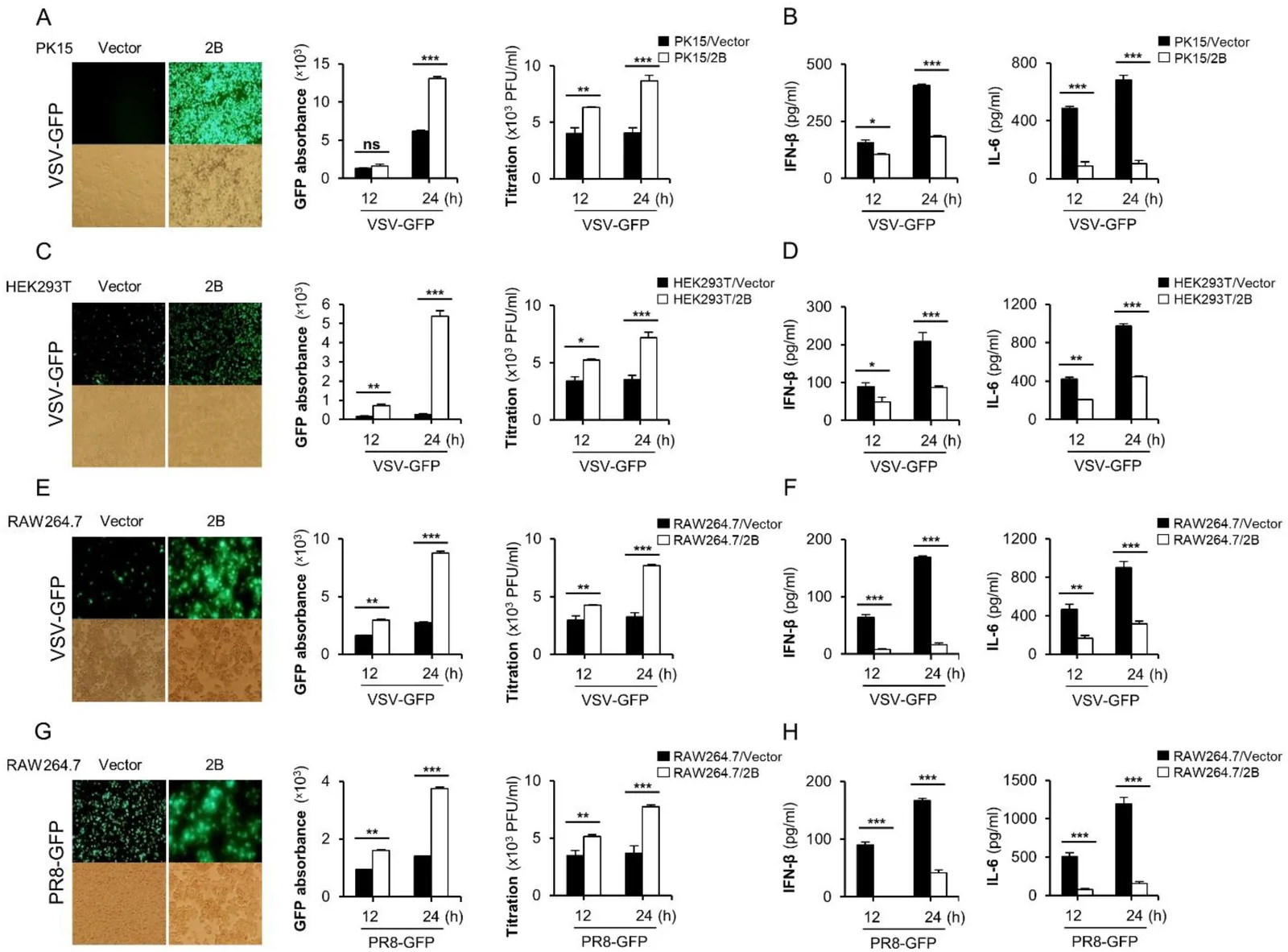
FMDV 2B negatively regulate RNA virus-mediated innate immune responses. PK-15 cells **(A, B)** and HEK293T cells **(C, D)** were transiently transfected with the control vector or FMDV 2B and infected with VSV-GFP. GFP expression, GFP absorbance, and virus titer was taken at 12 and 24 hpi **(A, C)**. The concentration of secreted IFN-β and IL-6 in supernatants was determined at 12 and 24 hpi by ELISA **(B, D)**. Control vector and FMDV 2B stably expressing RAW264.7 cells were infected with VSV-GFP **(E, F)** and PR8-GFP **(G, H)**. GFP expression, GFP absorbance, and virus titer was taken at 12 and 24 hpi **(E, G)**. The concentration of IFN-β and IL-6 secreted in supernatants were determined at 12 and 24 hpi by ELISA **(F, H)**. Results representative of at least two independent experiments, each with similar results, and the values are expressed as mean ± SD of three biological replicates. Student’s t-test; *p < 0.05; **p < 0.01; ***p < 0.001; ns, not significant.

Next, we investigated whether FMDV 2B affects the replication of other viruses. FMDV 2B expression plasmids were transiently transfected into porcine kidney epithelial cells (PK-15) and HEK293T cells (**Supplementary Figures 2A, B**) and were infected with GFP-tagged vesicular stomatitis virus (VSV-GFP) (**Figures 1A, C**), influenza A virus PR8 strain (PR8-GFP) (**Supplementary Figure 2D**), EV-71 (**Supplementary Figure 2H**), and coxsackievirus H3-GFP (**Supplementary Figure 2I**). Interestingly, we found that the virus replication in FMDV 2B-overexpressed cells was significantly higher than that in the control cells in different cell lines (**Figures 1A, C**). Next, we measured the amount of IFN-β and IL-6 secreted from the virus-infected cells using enzyme-linked immunosorbent assay (ELISA). Consistent with the results of the virus replication, we found that the FMDV 2B-overexpressed cells secreted fewer cytokines than the control cells (**Figures 1B, D**; **Supplementary Figure 2E**). Additionally, RAW264.7 cells stably expressing FMDV 2B were prepared (**Supplementary Figure 2C**) and infected with VSV-GFP (**Figure 1E**) and PR8-GFP (**Figure 1G**). As expected, a higher level of virus replication and lower levels of IL-6, IFN-β, IFN-α, TNF-α, and IL-1β secretions (**Figures 1E–H**, **Supplementary Figures 2F, G**) were observed in RAW264.7 cells stably expressing FMDV 2B. These results suggest that FMDV 2B negatively regulates the production of type-I IFNs and proinflammatory cytokines and enhances the RNA virus replication in the macrophage and the epithelial cell lines. These results suggest that FMDV 2B negatively regulates the type-I IFN pathway and weakens the antiviral status of the host cells by reducing the IFN secretion in response to a viral infection, thereby facilitating the RNA virus replication in the macrophage and the epithelial cell lines.

### FMDV 2B targets RIG-I and MDA5 to inhibit type-I IFN signaling

**Figure 2:**
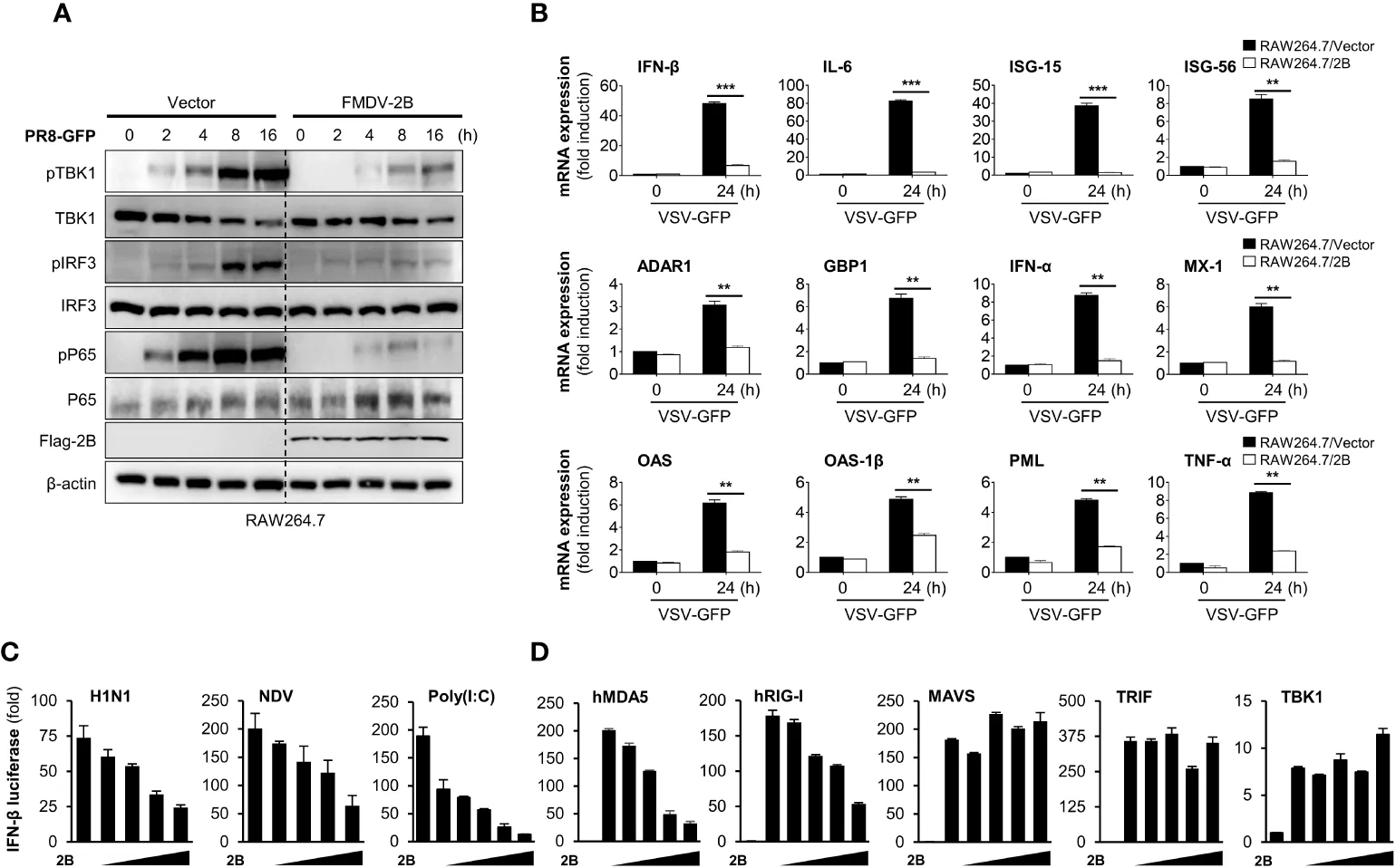
FMDV 2B inhibit the transcription of antiviral genes and type-I IFN signaling. Control vector and FMDV 2B stably expressing RAW264.7 cells were infected with PR8-GFP. Cells were harvested at indicated time points after the infection of PR8-GFP. Total and phosphorylated TBK1, IRF3, and p65 were measured by immunoblotting. β-actin was used as the loading control **(A)**. Control vector and FMDV 2B stably expressing RAW264.7 cells were infected with VSV-GFP. At 0 and 24 hpi, cells were harvested, and quantitative real-time PCR was performed to analyze the levels of antiviral genes **(B)**. HEK293T cells were transfected with interferon-β promoter encoding firefly luciferase plasmid, TK-Renilla plasmid, increasing dose of Flag-FMDV 2B plasmid and stimulated with H1N1, NDV infection, Poly(I:C) treatment **(C)** or transfected with MDA5, RIG-I, MAVS, TRIF, and TBK1 encoding plasmids **(D)** for 24 hours. Results are expressed relative to those of Renilla luciferase alone (internal control). Results representative of at least two independent experiments, each with similar results, and the values are expressed as mean ± SD of three biological replicates. Student’s t-test; **p < 0.01; ***p < 0.001.

To further confirm the effects of FMDV 2B on antiviral signaling cascades, we investigated RNA virus-mediated phosphorylation of TBK1, IRF3, and p65 in RAW264.7 cells stably expressing FMDV 2B. As shown in **Figure 2A**, phosphorylation levels of TBK1, IRF3, and p65 were significantly lower in FMDV 2B-overexpressed RAW264.7 cells than in the control cells at the indicated time points after the infection. Additionally, we also analyzed the effect of FMDV 2B on the transcription of IFNs and IFN-inducible genes in FMDV 2B-overexpressed RAW264.7 cells. For this experiment, we infected FMDV 2B-overexpressed RAW264.7 cells with VSV-GFP and performed real-time qPCR using specific primers. As a result, the expressions of mRNA encoding IFNs and other antiviral genes were significantly lower in FMDV 2B-overexpressed cells (**Figure 2B**) than they were in the control cells. These data support the notion that FMDV 2B negatively regulates the type-I IFN signaling pathway and expression of antiviral genes in response to a virus infection.

Next, to identify the potential target of FMDV 2B in the type-I IFN cascade, we performed a luciferase promoter assay by co-expressing both genes with several IFN-related genes as indicated in **Figures 2C, D**. We found that FMDV 2B markedly inhibited H1N1, NDV-GFP, poly(I:C), MDA5, and RIG-I-mediated activation of the IFN-β promoter in a dose-dependent manner (**Figures 2C, D**). However, no detectable changes occurred in MAVS, TRIF, and TBK1-mediated promoter activity with increased expression of FMDV 2B. These results suggested that FMDV 2B regulates type-I IFN signaling at the level of RIG-I and MDA5.

### FMDV 2B interacts with RIG-I and mediates the degradation of RIG-I

**Figure 3:**
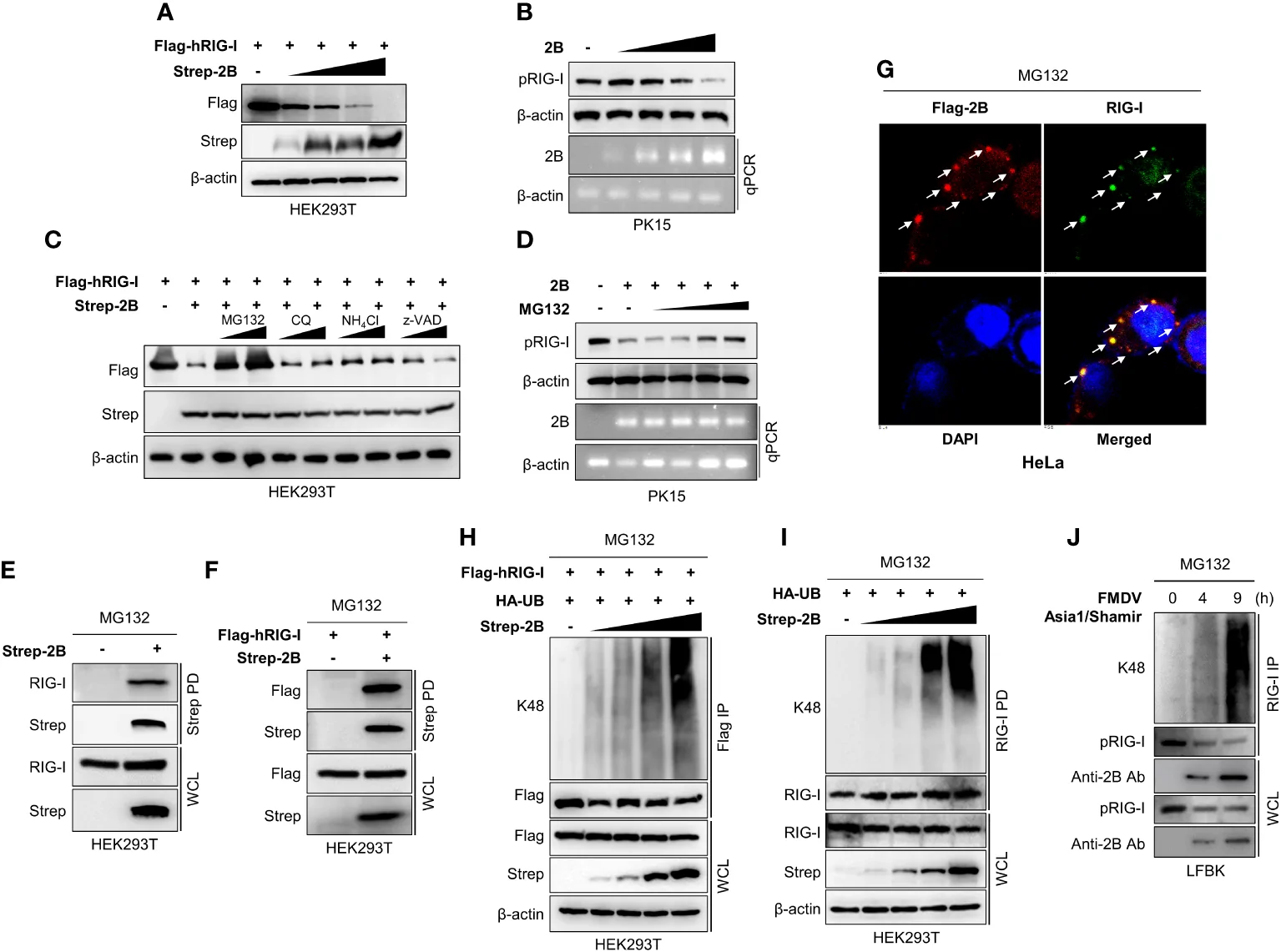
FMDV 2B targets RIG-I and mediates degradation of RIG-I *via* K48-linked polyubiquitination. HEK293T cells were transfected with Flag-hRIG-I plasmid, and increasing doses of Strep-FMDV 2B plasmids **(A)**, PK-15 cells were transfected with increasing amounts of FMDV 2B and 24 hrs post-transfection cells were infected with SeV [1 multiplicity of infection (MOI)] **(B)**. Cells were harvested at 24 hrs post-infection, and RIG-I expression level was measured by immunoblotting. HEK293T cells were transfected with Flag-hRIG-I together with Strep-FMDV 2B and treated with two doses of MG132, chloroquine, NH_4_Cl, or z-VAD for 6 hours before harvesting the cells. Whole-cell lysates were subjected to immunoblotting with indicated antibodies **(C)**. PK-15 cells were transfected with Strep-FMDV 2B, and 24 hrs post-transfection cells were infected with SeV (1MOI). Cells were treated with increasing doses of MG132 at 18 hours hrs post-infection and harvested at 24 hrs post-infection. The RIG-I expression level was measured by immunoblotting **(D)**. HEK293T cells were transfected with Strep-FMDV 2B **(E)** or Flag-RIG-I together with Strep-FMDV 2B **(F)** and treated with MG132 for 6 hours before harvesting the cells. Cell lysates were subjected to Strep pull-down and immunoblotted with the indicated antibodies. HeLa cells were transfected with Flag-FMDV 2B and treated with MG132 for 6 hours before fixing the plate, followed by confocal microscopy with anti-Flag (red) and anti-RIG-I (green) antibodies. Nuclei were stained with DAPI (blue) **(G)**. HEK293T cells were transfected with Flag-RIG-I, HA-ubiquitin together with increasing doses of Strep-FMDV 2B plasmids and treated with MG132 for 6 hours before harvesting the cells. Whole-cell lysates were subjected to Flag immunoprecipitation and immunoblotted with indicated antibodies **(H)**. HEK293T cells were transfected with HA-ubiquitin together with increasing doses of Strep-2B plasmids and treated with MG132 for 6 hours before harvesting the cells. Whole-cell lysates were subjected to immunoprecipitation with RIG-I antibody and immunoblotted with indicated antibodies **(I)**. LFBK cells were infected with FMDV Asia1/Shamir strain and treated with MG132 for 6 hours before harvesting the cells. Cells were harvested at indicated time after infection and whole-cell lysates were immunoprecipitated with anti-RIG-I antibody and immunoblotted with indicated antibodies **(J)**. Results representative of at least two independent experiments, each with similar results. (pRIG-I: porcine RIG-I, hRIG-I: human RIG-I).

Many viruses express proteins that target RIG-I to suppress the host defense mechanisms. A lot of the interactions between viral proteins and RIG-I result in the cleavage, degradation, suspension, or inhibition of RIG-I (16). Based on our luciferase results, FMDV 2B targets RIG-I, and previous reports have also shown that FMDV 2B inhibits RIG-I expression (23, 27). In this study, we first investigated the degradation of RIG-I by FMDV 2B. HEK293T cells were co-transfected with RIG-I expression plasmids and increasing doses of FMDV 2B expression plasmids, followed by an immunoblot analysis of whole-cell lysates. Interestingly, we found that the overexpression of FMDV 2B resulted in a significant decrease in RIG-I levels, which was FMDV 2B dose-dependent (**Figure 3A**). Concurrently, we confirmed that FMDV 2B reduced the expression of the endogenous porcine RIG-I (pRIG-I) in PK-15 cells in a dose-dependent manner (**Figure 3B**).

Next, to determine whether FMDV 2B affects the expression or the degradation of RIG-I, we co-transfected RIG-I and FMDV 2B expression plasmids into cells treated with different inhibitors to determine the type of degradation caused by FMDV 2B. As shown in **Figure 3C, D**, the expression of RIG-I was restored upon treatment with a proteasomal inhibitor MG132 but not upon treatment with lysosomal inhibitors (chloroquine and NH_4_Cl) or a caspase inhibitor (z-VAD). These results suggest that RIG-I undergoes FMDV 2B-mediated proteasomal degradation. Similarly, FMDV 2B also degrades RIG-I 2CARD (1-186 aa fragment of RIG-I containing CARD1 and CARD2 domains) (**Supplementary Figure 3B**) but does not induce RIG-I or RIG-I 2CARD degradation in the presence of MG132 (**Supplementary Figures 3A, C**). Next, we performed an immunoprecipitation assay to assess whether FMDV 2B physically interacts with RIG-I and induces degradation. As shown in **Figures 3E, F**, FMDV 2B strongly interacted with RIG-I in the presence of MG132, and additionally, we confirmed the co-localization of FMDV 2B and RIG-I with MG132 treatment (**Figure 3G**). Thus, we next asked whether RIG-I degradation is mediated by ubiquitination. As shown in **Figure 3H**, HEK293T cells were co-transfected with the indicated plasmids in the presence of MG132, followed by immunoprecipitation and immunoblotting with a K48-specific antibody. Interestingly, the RIG-I underwent K48-linked ubiquitination in the presence of FMDV 2B, and its ubiquitination activity was enhanced by increasing doses of FMDV 2B. K48 ubiquitination of the endogenous RIG-I by FMDV 2B was also examined (**Figure 3I**). Next, to further validate whether RIG-I undergo ubiquitination upon FMDV infection, we infected the fetal porcine kidney (LFBK) cells with FMDV in the presence of MG132. Results of **Figure 3J** shows K48 ubiquitination of RIG-I and ubiquitination enhanced with time after virus infection. These findings indicate that FMDV 2B mediates ubiquitin-dependent proteasomal degradation of RIG-I by conjugating K48-linked polyubiquitin chains.

### FMDV 2B recruits RNF125 to mediate degradation of RIG-I by K48 ubiquitination

**Figure 4:**
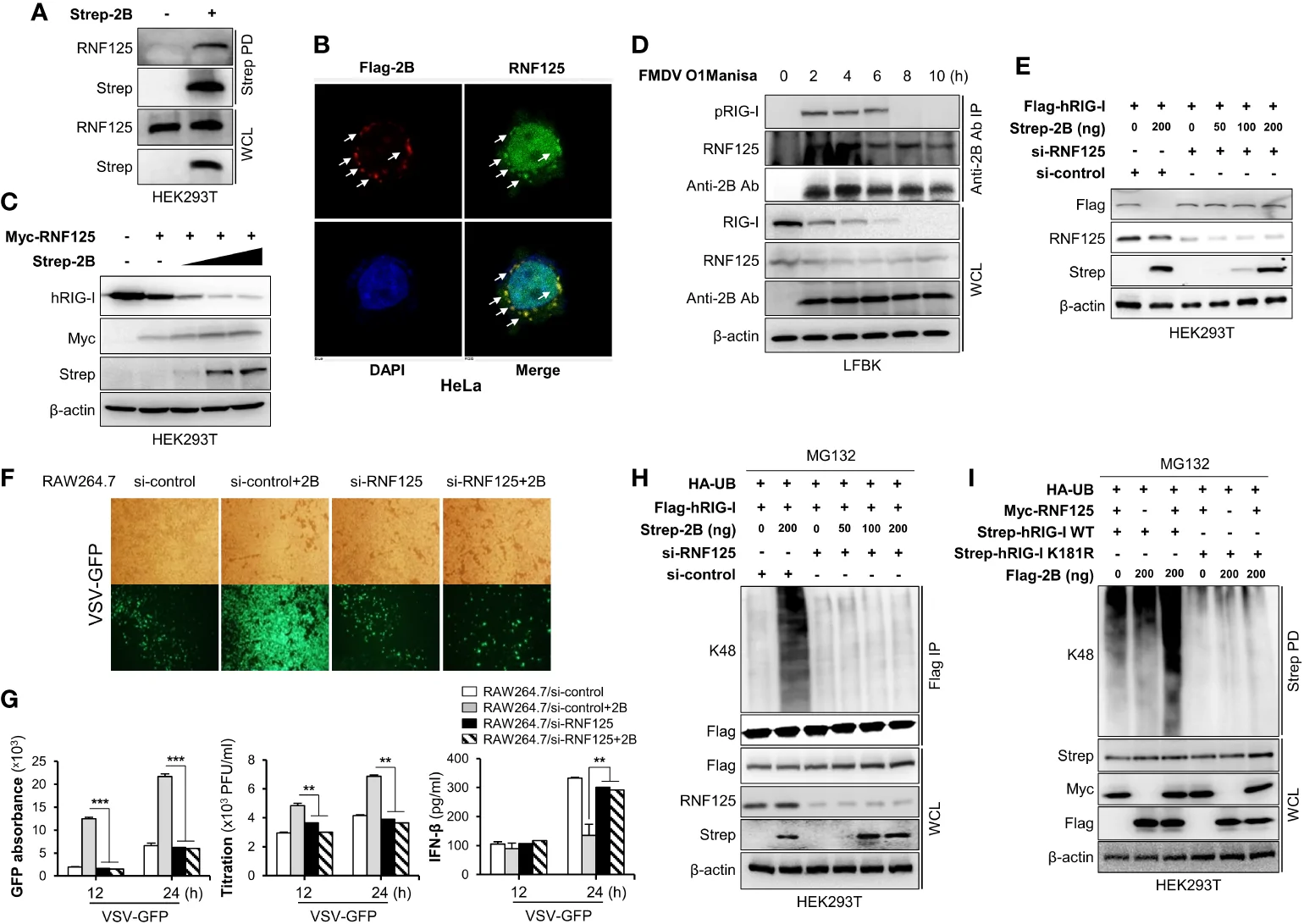
FMDV 2B recruit RNF125 to mediate K48-linked polyubiquitination and degradation of RIG-I. HEK293T cells were transfected with Strep-FMDV 2B. Cell lysates were subjected to Strep pull-down assay and immunoblotted with indicated antibodies **(A)**. HeLa cells were transfected with Flag-FMDV 2B, followed by confocal microscopy assay with anti-Flag (red) and anti-RNF125 (green) antibodies. Nuclei were stained with DAPI (blue) **(B)**. HEK293T cells were transfected with Myc-RNF125 together with increasing doses of Strep-FMDV 2B. The whole-cell lysate was immunoblotted with indicated antibodies **(C)**. LFBK cells were infected with FMDV O1Manisa strain, and cells were harvested after indicated time period post-infection. The whole-cell lysate was immunoprecipitated with FMDV 2B rabbit polyclonal antibody and immunoblotted with indicated antibodies **(D)**. HEK293T cells were transfected with si-control or si-RNF125 together with Flag-RIG-I and different doses of Strep-2B plasmids. The whole-cell lysate was immunoblotted with indicated antibodies **(E)**. **(F, G)** RAW264.7 cells were transfected with si-control or si-RNF125 together with Flag-FMDV 2B and infected with VSV-GFP. Viral replication was measured by fluorescence microscopy, GFP absorbance, and plaque assay at indicated time points. The concentration of secreted IFN-β in supernatants was determined at 12 and 24 hpi by ELISA. HEK293T cells were transfected with si-control or si-RNF125 together with Flag-RIG-I, HA-ubiquitin, indicated doses of Strep-FMDV 2B and treated with MG132 6 hours before harvesting the cells. Whole-cell lysates were subjected to Flag-immunoprecipitation and immunoblotted with indicated antibodies **(H)**. HEK293T cells were transfected with Strep-RIG-I wild-type or RIG-I K181R mutant together with HA-ubiquitin, Myc-RNF-125, indicated doses of Flag-FMDV 2B and treated with MG132 6 hours before harvesting the cells. Whole-cell lysates were subjected to Strep pull-down and immunoblotted with indicated antibodies **(I)**. Results representative of at least two independent experiments, each with similar results, and the values are expressed as mean ± SD of three biological replicates. Student’s t-test; **p < 0.01; ***p < 0.001.

While some viruses have non-canonical E3 ligases (31), others use alternative strategies to maintain E3 ligase activity by recruiting and redirecting host E3 ligases for the ubiquitination of the host proteins. For example, the E6 oncoproteins of human papillomavirus (HPV) induce p53 degradation by recruiting host E3 ligase E6-associated protein (E6AP) (32–34). Since FMDV 2B does not have E3 ligase activity on its own, we hypothesized that FMDV 2B recruits host E3 ligase for the ubiquitination and degradation of RIG-I. Hence, we searched for a potential E3 ligase that mediates the proteasomal degradation of RIG-I and selected RNF125. RNF125 is the first identified E3 ligase that conjugates K48-linked ubiquitination and mediates the degradation of RIG-I *via* the proteasome pathway (35). To determine whether FMDV 2B interacts with RNF125, HEK293T cells were transfected with the indicated plasmids and a co-IP assay was performed. Interestingly, we found that the RNF125 was precipitated from the lysate of cells transfected with the FMDV 2B expression plasmids (**Figure 4A** and **Supplementary Figure 4A**). Consistent with the above results, we confirmed the co-localization of the RNF125 and FMDV 2B by confocal microscopy (**Figure 4B**).

Next, to identify the mechanism underlying the RNF125-mediated degradation of RIG-I, we set out to investigate whether RNF125 degrades RIG-I in the presence of increasing doses of an FMDV 2B expression plasmid. As expected, the expression of RIG-I was reduced by RNF125 (**Figure 4C**). Interestingly, RNF125 increased the degradation of RIG-I in an FMDV 2B dose-dependent manner under endogenous (**Figure 4C**) and overexpressed conditions (**Supplementary Figure 4B**).

Next, to investigate RIG-I status during FMDV infections, LFBK cells were infected with FMD O1 Manisa virus, and RIG-I levels were measured over time. Cell lysates were inactivated and immunoprecipitated with an anti-FMDV 2B antibody, then immunoblotted with anti-RIG-I and anti-RNF125 antibodies. As shown in **Figure 4D**, the FMDV 2B interacted with the RIG-I and the RNF125. Interestingly, at 6 hours post-infection (hpi), the overall amounts of the RIG-I began to decrease, and at 8 hpi, the interaction between the RIG-I almost disappeared. These results indicate that FMDV induces marked degradation of RIG-I in an RNF125-mediated manner as the infection progresses.

To further evaluate whether RNF125 is essential for FMDV 2B-mediated degradation of RIG-I, we designed siRNA targeting RNF125 and performed an immunoblotting to detect the RIG-I expression after the overexpression of the indicated plasmids in the RNF125 knockdown cells or the control HEK293T cells. As shown in **Figure 4E**, the knockdown of RNF125 did not promote RIG-I degradation. These results suggest that the degradation of RIG-I by FMDV 2B is dependent on RNF125. To confirm these results, we performed virus replication experiments in which RAW264.7 cells were treated with control or RNF125 siRNA, then transfected with the indicated plasmids, and lastly, infected with VSV-GFP. As shown in **Figures 4F, G**, the knockdown of RNF125 did not induce an enhancement of viral replication and inhibition of IFN-β secretion. Furthermore, to reassess whether RNF125 is essential for K48-linked polyubiquitination of RIG-I, the RNF125 knockdown or the control HEK293T cells were transfected with the indicated plasmids, and whole cell lysates were immunoprecipitated with Flag-tagged RIG-I. As shown in **Figure 4H**, the knockdown of RNF125 failed to promote K48-linked ubiquitination of RIG-I. Previous studies suggest that the lysine residue at position 181 of RIG-I is essential for RNF125-mediated ubiquitination and degradation of RIG-I (35). Hence, we constructed the RIG-I K181R mutant to confirm the hypothesis that FMDV 2B degrades RIG-I by recruiting RNF125. As shown in **Figure 4I** and **Supplementary Figure 4C** the RIG-I undergoes ubiquitination in the presence of RNF125 and FMDV 2B, whereas the RIG-I K181R mutant does not. Taken together, these results indicate that FMDV 2B hijacks the host RIG-I degradation mechanism mediated by RNF125 to suppress the host immune response to FMDV infection.

### C-terminal region of FMDV 2B is essential for the interaction and proteasomal degradation of RIG-I

**Figure 5:**
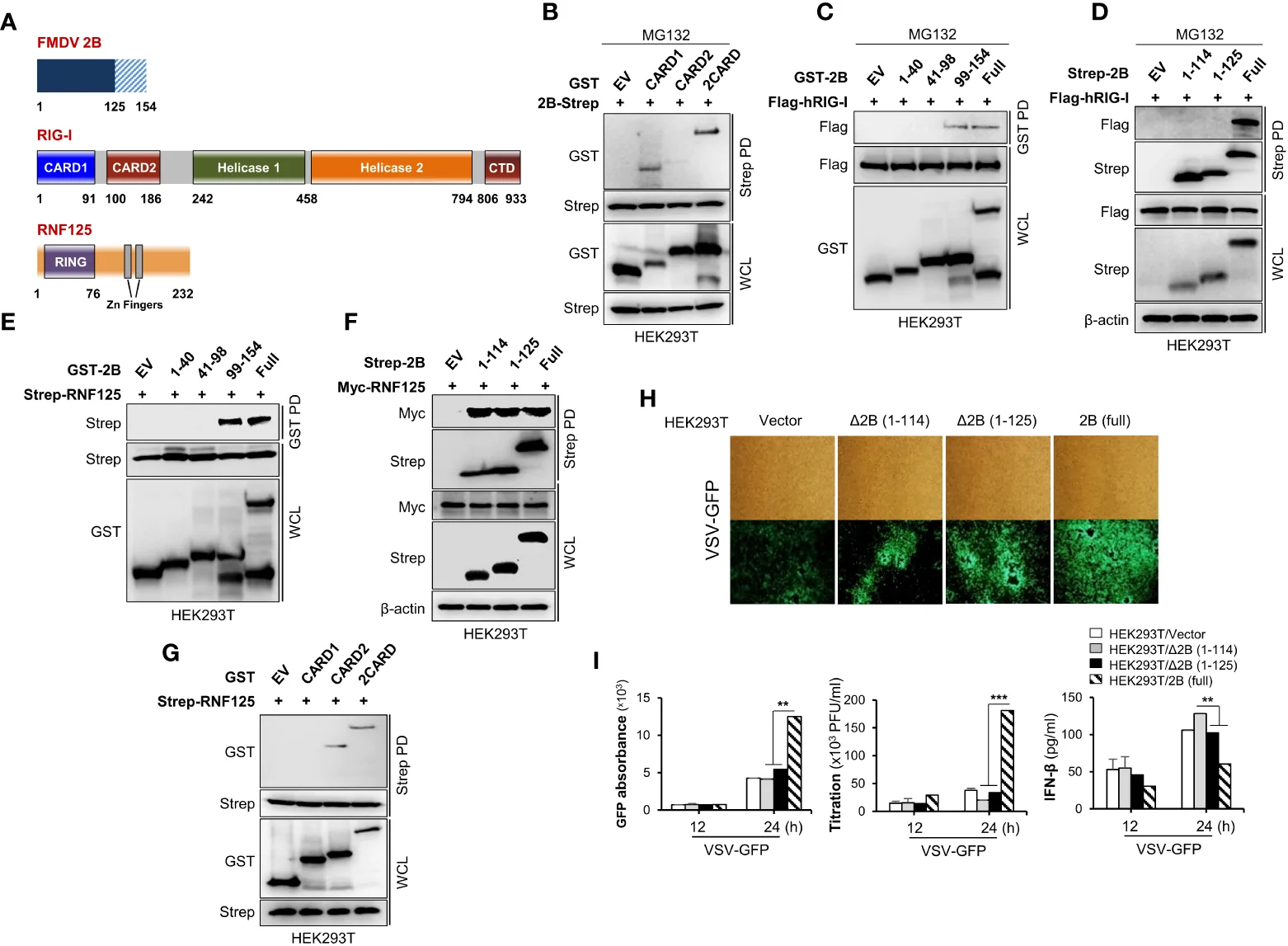
C-terminal region of FMDV 2B is important for the interaction and proteasomal degradation of RIG-I. Schematic representation of the domain construction of FMDV 2B, RIG-I, and RNF125 **(A)**. HEK293T cells were transfected with GST-RIG-I domain constructs together with Strep-FMDV 2B plasmids and treated with MG132 for 6 hours before harvesting the cells. Whole-cell lysates were subjected to the Strep pull-down assay, followed by immunoblotting with indicated antibodies **(B)**. HEK293T cells were transfected with Flag-RIG-I together with GST-FMDV 2B domain constructs containing plasmids and treated with MG132 for 6 hours before harvesting the cells. Whole-cell lysates were subjected to GST pull-down and immunoblotted with indicated antibodies **(C)**. HEK293T cells were transfected with Strep-FMDV 2B domain constructs together with Flag-RIG-I plasmids and treated with MG132 for 6 hours before harvesting the cells. Whole-cell lysates were subjected to the Strep pull-down assay, followed by immunoblotting with indicated antibodies **(D)**. HEK293T cells were transfected with Strep-RNF125 together with GST-FMDV 2B domain constructs containing plasmids. Whole-cell lysates were subjected to GST pull own and immunoblotted with indicated antibodies **(E)**. HEK293T cells were transfected with Myc-RNF125 together with Strep-FMDV 2B deletion mutant constructs containing plasmids. Whole-cell lysates were subjected to Strep pull own and immunoblotted with indicated antibodies **(F)**. HEK293T cells were transfected with Strep-RNF125 together with GST-RIG-I domain constructs containing plasmids. Whole-cell lysates were subjected to strep pull down and immunoblotted with indicated antibodies **(G)**. **(H, I)** HEK293T cells were transfected with control vector, FMDV Δ2B (1-114) mutant, FMDV Δ2B (1-125) mutant, and FMDV 2B wild-type plasmids and infected with VSV-GFP. Viral replication was measured by fluorescence microscopy, GFP absorbance, and plaque assay at indicated time points. The concentration of secreted IFN-β in supernatants was determined at 12 and 24 hpi by ELISA. Results representative of at least two independent experiments, each with similar results, and the values are expressed as mean ± SD of three biological replicates. Student’s t-test; **p < 0.01; ***p < 0.001.

While our experiments confirmed an interaction between RIG-I and FMDV 2B, the specific domains mediating this association were still unclear. To identify the domain within RIG-I that interacts with FMDV 2B, we constructed GST-fused CARD1, CARD2, or 2CARD of RIG-I expression plasmids (**Figure 5A**) and performed an immunoprecipitation assay. As shown in **Figure 5B**, the FMDV 2B bound strongly to the CARD1 (aa 1-92) of RIG-I. Similarly, a series of truncated mutants of FMDV 2B were constructed, and a GST pull-down assay using these mutants was performed. As shown in **Figures 5C, D**, RIG-I interacted with C-terminal amino acids (aa) 126-154 of FMDV 2B. Next, we investigated which region of FMDV 2B was required for the interaction with RNF125. As shown in **Figures 5E, F**, aa 99-113 of FMDV 2B is involve in the interaction between FMDV 2B and RNF125. We also reconfirmed the interaction between CARD2 (aa 93-186) of RIG-I and RNF125 as in previous studies (35) (**Figure 5G**). These results suggest that the aa 99-113 region of FMDV 2B recruits RNF125 and the aa 126-154 region interacts with RIG-I for RNF125-mediated ubiquitination and degradation of RIG-I. Based on these findings, to investigate whether the C-terminal deletion of FMDV 2B affects the RIG-I-mediated type-I IFN cascade, we generated two FMDV deletion mutants containing aa 115-154 deleted FMDV△2B (1–114) and aa 126-154 deleted FMDV△2B (1–125). As shown in **Figures 5H, I**, each indicating plasmid was transiently transfected into HEK293T cells, and each cell was infected with VSV-GFP. Interestingly, we found that the deletion mutants of FMDV 2B did not show higher levels of virus replication and lower levels of IFN-β secretion as in FMDV 2B. Altogether, these results indicated that the aa 126-154 region of FMDV 2B was essential for interaction with RIG-I, and this interaction was crucial for FMDV 2B-mediated inhibition of type-I IFN cascade *via* the ubiquitination and proteasomal degradation of RIG-I.

### FMDV 2B induces apoptosis and mediates caspase-3- and caspase-8-dependent degradation of MDA5

**Figure 6:**
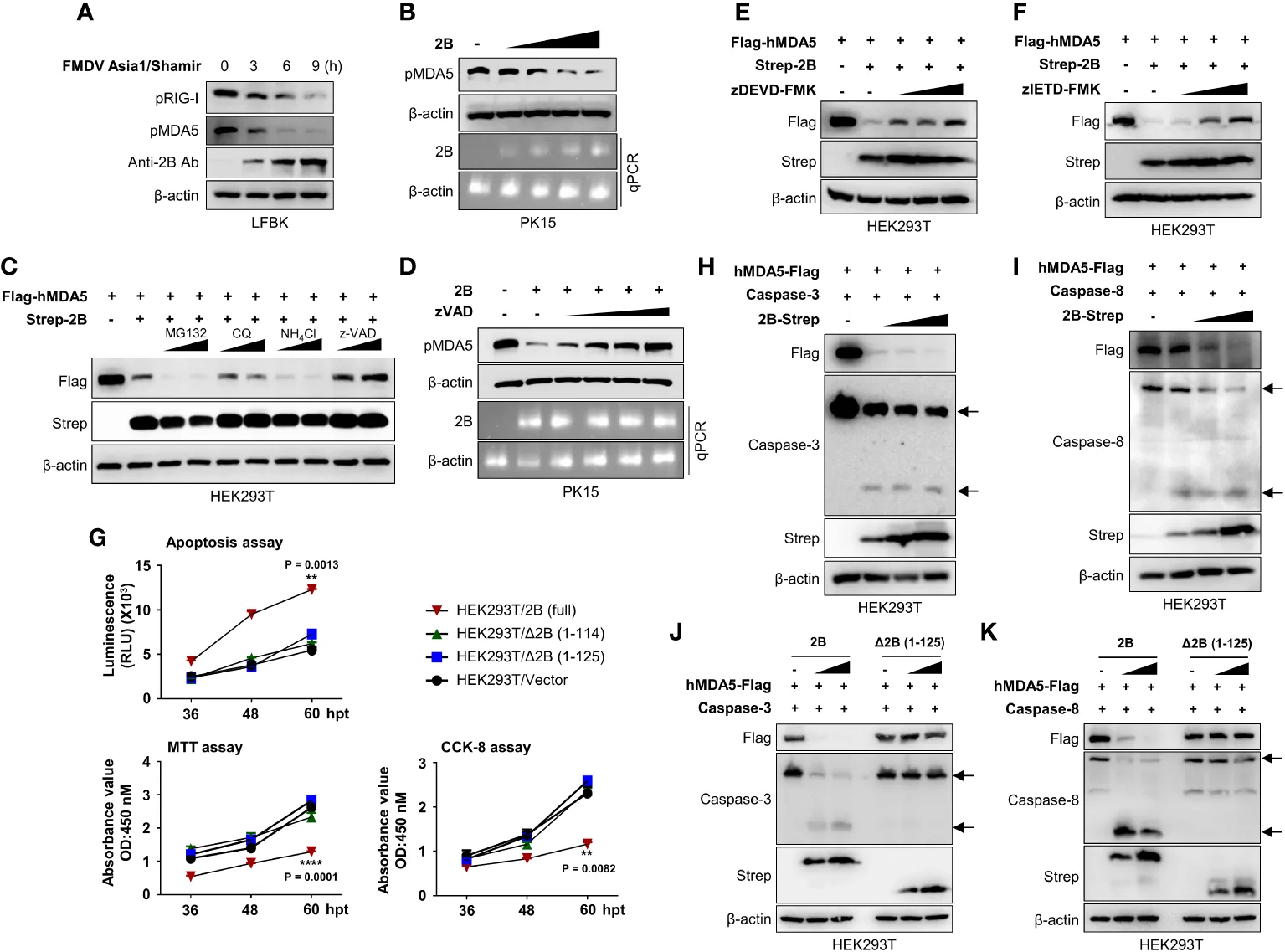
FMDV 2B induces apoptosis and apoptosis-mediated caspase-3 and caspase-8 dependent degradation of MDA5. LFBK cells were infected with 0.1MOI of FMDV Asia1/Shamir for 0, 3, 6, 9 hours and whole-cell lysates were immunoblotted with indicated antibodies **(A)**. PK-15 cells were transfected with increasing doses of FMDV 2B, and 24 hrs post-transfection cells were infected with SeV (1MOI). Cells were harvested at 24 hrs post-infection, and the MDA5 expression level was measured by immunoblotting **(B)**. HEK293T cells were transfected with Flag-MDA5 together with Strep-FMDV 2B and treated with two doses of MG132, chloroquine, NH_4_Cl, or zVAD for 6 hours before harvesting the cells. Whole-cell lysates were subjected to immunoblotting with indicated antibodies **(C)**. PK-15 cells were transfected with FMDV 2B, and 24 hrs post-transfection, cells were infected with SeV (1MOI). Cells were treated with increasing doses of zVED for 6 hours before harvesting cells. Whole-cell lysates were subjected to immunoblotting with indicated antibodies **(D)**. **(E, F)** HEK293T cells were treated with Flag-hMDA5, Strep-FMDV 2B plasmids, and treated with increasing doses of zDEVD-FMK **(E)**, or zIETD-FMK **(F)**, 6 hours before harvesting the cells. Whole-cell lysates were subjected to immunoblotting with indicated antibodies. HEK293T cells were transfected with Strep-FMDV 2B wild-type, FMDV Δ2B (1-114) mutant, FMDV Δ2B (1-125) mutant, and control vector. Annexin V apoptosis assay, CCK-8 assay, and MTT assay were conducted at indicated time points post-transfection **(G)**. **(H, I)** HEK293T cells were transfected with Flag-MDA5, caspase-3 **(G)**, or caspase-8 **(H)** plasmids together with increasing doses of Strep-FMDV 2B plasmids. Whole-cell lysates were subjected to immunoblotting with indicated antibodies. **(J, K)** HEK293T cells were transfected with Flag-MDA5, caspase-3 **(J)**, or caspase-8 **(K)** plasmids together with increasing doses of Strep-FMDV 2B wild-type or FMDV Δ2B (1-125) mutant. Whole-cell lysates were subjected to immunoblotting with indicated antibodies. Results representative of at least two independent experiments, each with similar results. Student’s t-test; **p < 0.01; ****p < 0.0001.

Cytosolic RNA sensor MDA5 is known to be essential for the recognition of FMDV (36). Hence we checked the effect of FMDV infection on MDA5 protein expression. Results of **Figure 6A** shows that FMDV infection in to LFBK cells reduced the MDA5 protein expression similar to RIG-I (**Figure 6A**; **Supplementary Figure 5A**). Based on the IFN-β reporter assay (**Figure 2D**) and the results of previous studies (27), we also investigated whether FMDV 2B affects MDA5. First, HEK293T cells were co-transfected with MDA5 expression plasmids and increasing doses of FMDV 2B expression plasmids, followed by an immunoblot analysis. As shown in **Supplementary Figure 5B**, we found that the overexpression of FMDV 2B resulted in a significant decrease in the MDA5 levels in a dose-dependent manner. Concurrently, we confirmed that FMDV 2B also reduced the expression of the endogenous porcine MDA5 (pMDA5) in PK-15 cells in a dose-dependent manner (**Figure 6B**). Next, to confirm whether FMDV 2B affects the expression or the degradation of MDA5, we co-transfected FMDV 2B and MDA5 expression plasmids into cells treated with different inhibitors. As shown in **Figure 6C**, the expression of MDA5 was restored upon treatment with a z-VAD. In addition, the degradation of the endogenous MDA5 in PK-15 cells was recovered in a dose-dependent manner by treatment with z-VAD (**Figure 6D**).

In the previous studies, mouse MDA5 undergoes caspase-3- and caspase-8-dependent cleavage upon apoptosis stimulation (18). To investigate whether the FMDV 2B-mediated degradation of MDA5 is caspase-3- or caspase-8-dependent, HEK293T cells were co-transfected with MDA5 and FMDV 2B expression plasmids in the presence of increasing doses of a caspase-3 inhibitor (zDEVD-FMK) or a caspase-8 inhibitor (zIETD-FMK). As shown in **Figures 6E, F**, the MDA5 was not degraded in the presence of both inhibitors.

Picornaviruses induce cellular apoptosis after infection (37). Among the various viral proteins of picornaviruses, viroporins modify membrane permeability and disrupt the Ca^2+^ balance, leading to apoptosis (26, 38, 39). In particular, viroporin is known to induce caspase-dependent apoptosis (39). Since FMDV 2B shows viroporin activity (26), we investigated the apoptotic activity of FMDV 2B using an Annexin V apoptosis assay, a cell counting kit-8 (CCK-8) assay, and 3-[4,5-dimethylthiazol-2-yl]-2,5-diphenyltetrazolium bromide (MTT) assay. As shown in **Figure 6G** and **Supplementary Figure 5C**, FMDV 2B induces significant apoptosis in HEK293T cells and PK-15 cells after transfection with FMDV 2B expression plasmids. However, surprisingly, FMDV△2B (1-114) and FMDV△2B (1-125) did not show apoptosis. Next, we checked the activation of caspase-3 and caspase-8 in the presence of increasing doses of FMDV 2B. As shown in **Figure 6H, I**, the FMDV 2B activated both caspase-3 and caspase-8, and activation of these caspases was consistent with the degradation of the MDA5. However, FMDV△2B (1-114) and FMDV△2B (1-125) neither activated the caspases nor degraded the MDA5 (**Figures 6J, K**; **Supplementary Figures 5D, E**). Taken together, these results suggest that the C-terminal region of FMDV 2B is critical for the induction of caspase-dependent apoptosis and the degradation of MDA5 in a dose-dependent manner. Consequently, FMDV 2B affects the MDA5-mediated type-I IFN cascade.

## Discussion

The RLR-mediated Type-I IFN response is a critical defense mechanism against RNA viruses, including FMDV (16). Therefore, FMDV is highly sensitive to the IFN response. To avoid this, FMDV has evolved various immune evasion strategies to ensure effective replication in host cells (40). FMDV proteases, L^pro^, and 3C^pro^ play a crucial role in interfering with host protein translation to induce FMDV replication within host cells. Specifically, FMDV L^pro^ is associated with cleavage of translation initiation factor eIF4G to inhibit cellular protein synthesis (41), mediate the degradation of NF-кB subunit, p65/RelA (42), inhibition of NF-кB dependent gene expression (43), reduced expression of IFN-β mRNA levels by direct cleavage of LGP2 (44), acting as a viral deubiquitinase to deubiquitinate RIG-I, TBK1, TRAF3, and TRAF6 (45), to enhance FMDV replication by suppressing host immune response. 3C^pro^ has mainly involved in the inhibition of NF-кB and IRF signaling, followed by direct cleavage of NEMO (46), cleavage of nuclear protein Sam68 (47), cleavage of G3BP1 and inhibit stress granule formation (48), degradation of KPNA1 nuclear translocation signal receptor to inhibit STAT1/STAT2 nuclear translocation (49), degradation of cellular viral RNA sensors, RIG-I, MDA5 (50), and LGP2 (30). Apart from proteases, other FMDV non-structural proteins also play a significant role in modulating host immune response. FMDV 2C is associated with inhibition of autophagy and enhanced viral replication by interacting with Beclin-1 (51). FMDV 3A is the largest 3A protein among all picornavirus family members. It is also associated with disruption of RLR-mediated IFN-β signaling by downregulating RIG-I, MDA5 transcript levels (52), DDX56 dependent inhibition of IRF3 phosphorylation (53), upregulation of LRRC25 to inhibit G3BP1 mediated RLH signaling pathway (9). FMDV VP3 mediates the degradation of JAK1 (54), decreases the expression of RIG-I and MDA5 (55) and inhibits MAVS aggregation (56). Furthermore, FMDV VP1 interacts with sorcin to inhibit IFN signaling (57). Moreover, a recent study shows that FMDV VP1 inhibits MAVS-TRAF3 binding to downregulate interferon signaling and VP1 E83K mutated virus shows attenuation in pigs (58).

In this study, we demonstrated two novel molecular mechanisms of FMDV 2B-mediated RIG-I and MDA5 degradation to evade host IFN responses. First, we showed that the overexpression of FMDV 2B in epithelial cells induces enhancement of RNA virus replication and also downregulates RNA virus-induced IFN-β and proinflammatory cytokine-signaling cascades. Second, FMDV 2B interacted with RIG-I and induced K48 ubiquitination and proteasomal degradation of the RIG-I by recruiting E3 ubiquitin ligase, RNF125. Third, FMDV 2B protein was involved in inducing apoptosis and apoptosis-mediated caspase-3- and caspase-8-dependent cleavage of MDA5. Fourth, aa 126-154 of FMDV 2B are essential for 2B-mediated degradation of RIG-I and MDA5. Taken together, these findings indicate a specific molecular mechanism of FMDV 2B that negatively regulates host type-I IFN signaling by degradation of RIG-I and caspase-dependent degradation of MDA5.

FMDV 2B is known to be primarily associated with host cell membrane rearrangement and the inhibition of the cellular secretory pathway (ER-to-Golgi transport) (24, 40). Sequence and structural analysis of FMDV 2B showed that the 2B protein consists of two hydrophobic regions and is mainly localized in ER (26, 40). Previous studies have shown that FMDV 2B increases Ca^2+^ ion content in host cells and also increases membrane permeability in both bacteria and mammalian cells (26). Consequently, researchers suggest that FMDV 2B acts as a viroporin during viral infections, and it is also known that the viroporin activity of FMDV 2B mediates NLRP3 inflammasome activation (59). Notably, it has already been suggested that FMDV 2B is involved in the impairment of RIG-I or LGP2-mediated antiviral signaling (23, 30), and Zhu and colleagues suggested that FMDV 2B inhibits the expression of RIG-I and MDA5 to antagonize RIG-I-mediated type-I IFN response (23). However, the precise molecular mechanisms of FMDV 2B targeting RIG-I and MDA5 for negative regulation of type-I IFN signaling is still unknown. In this study, we showed an IFN inhibition phenotype after the overexpression of FMDV 2B as in previous reports and confirmed that FMDV 2B targets RIG-I by an IFN-β luciferase reporter assay. However, we evaluated the effect of MG132 on HEK293T cell overexpression system (**Figure 3C** and **Supplementary Figure 3A**), as well as in PK15 cell endogenous system (**Figure 3D**), showing a clear inhibition of 2B-mediated RIG-I degradation by MG132, which differs from the previous report. Ultimately, we found an interaction between FMDV 2B and RIG-I and demonstrated the degradation of RIG-I rather than the inhibition of protein expression.

The overexpression of FMDV 2B significantly induced the degradation of RIG-I in a dose-dependent manner, and its degradation was found to be proteasomal by K48 ubiquitination. Although FMDV 2B can induce K48 ubiquitination of RIG-I, it does not have E3 ligase activity. Therefore, we hypothesized that 2B enhances ubiquitination and degradation of RIG-I by recruiting a host E3 ligase such as a latent membrane protein 1 (LMP1) of Epstein-Barr virus (EBV), which mediates the proteasomal degradation of RIG-I by recruiting E3 ligase CHIP (60). So far, RNF125, CHIP, RNF122, and MARCH5 have been reported to mediate the K48 ubiquitination and the degradation of RIG-I for the downregulation of RIG-I-mediated IFN signaling (35, 61–63). Among them, we found that FMDV 2B specifically interacted with RNF125 and strongly co-localized in HeLa cells, and this interaction increases proteasomal degradation followed by K48 ubiquitination of RIG-I (**Figures 4B, C, H**). Previous studies have reported that the K181 residue of the RIG-I is responsible for the ubiquitination and degradation by RNF125 (35). Therefore, for more reliable evidence, we tested the effect of a (RIG-I) (K181R) mutant on FMDV 2B-mediated RIG-I ubiquitination, and showed that the FMDV 2B was unable to induce the ubiquitination and degradation of RIG-I (K181R) by RNF125 (**Figure 4I**). Furthermore, we also found that the overexpression of FMDV 2B significantly induced the degradation of MDA5 in a dose-dependent manner. We further investigated the mechanism of the MDA5 degradation by FMDV 2B and found that it is a caspase-dependent degradation. However, we could not see an interaction between FMDV 2B and MDA5 in the presence of the caspase inhibitors. Therefore, we hypothesized that this degradation of MDA5 was a binding-independent degradation. Previously, Kovacsovics and colleagues demonstrated that the MDA5 undergoes caspase-dependent cleavage upon stimulation of apoptosis and that this cleavage of MDA5 is a caspase-3- or caspase-8-dependent cleavage that can be inhibited by specific inhibitors (DEVD, IETD) (18). Furthermore, previous findings have shown that infections of picornaviruses, such as Enterovirus 71 (EV71) and Poliovirus (PV), sequentially induce apoptosis and the cleavage of MDA5 without proteinases (2A^pro^, 3C^pro^) activity (64). In this study, together with previous findings, we showed that the overexpression of FMDV 2B activates the apoptotic pathway. The apoptotic stimulation converted procaspase-3 and procaspase-8 into an activated form, and as a result, caspase-3- or caspase-8-dependent degradation of MDA5 was confirmed (**Figures 6H, I**).

Based on the mapping study, we identified that the C-terminal fragment (aa 126-154) of 2B is essential for the interaction between CARD1 domain of RIG-I and FMDV 2B (**Figures 5B, C**). For the impact of the interaction domain of 2B, we constructed 2B mutant △126-154 and found that the mutant was unable to induce the ubiquitination and the degradation of RIG-I and ultimately lost the ability to modulate the IFN response (**Figures 5D, H, I**). Interestingly, the mutant was also unable to induce apoptosis leading to caspase-dependent cleavage of MDA5 (**Figures 6G–K**). Additionally, we confirmed that the C-terminal domain (aa 99-114) of 2B interacts with RNF125, and RNF125 interacts with CARD2 domain of RIG-I individually without interfering with complex formations (**Figures 5E–G**).

Currently, there are commercially available vaccines against FMDV. Nevertheless, the research and necessity for attenuated FMDV strains are required in various aspects including safety. The development of the attenuated FMDV strain by genetic manipulation is a reasonable approach and, perhaps, a safer methodology. To confirm the role and the molecular mechanism of FMDV 2B identified in the FMDV itself, a construction of a recombinant FMD virus harboring 2B mutation (△126-154) and an evaluation of virulence in swine are needed in the future.

In summary, ongoing studies of the immune evasion mechanisms used by FMDV are critical to better understanding the pathogenesis of FMDV. Our results demonstrate that FMDV 2B is a negative regulator of type-I IFN-signaling cascade for virus replication. To inhibit type-I IFN-signaling, FMDV 2B recruits the E3 ubiquitin ligase RNF125 to induce proteasomal degradation of RIG-I by K48 ubiquitination and also targets MDA5 for caspase-3- and caspase-8-dependent cleavage of the MDA5 by apoptosis induction. These findings may provide a new understanding of molecular mechanisms used by the FMDV 2B to counteract the type-I IFN responses and expand our knowledge on immune evasion strategies used by FMDV. Furthermore, our study may provide a rational approach to virus attenuation for future FMDV vaccine development.

## Materials and methods

### Cells and antibodies

RAW264.7 (ATCC TIB-71), HEK293T (ATCC-11268), HeLa (ATCC CCL-2), PK-15 (ATCC CCL-33), LFBK (RRID:CVCL_RX26), cells were cultured in Dulbecco’s Modified Eagle Medium (DMEM) (HyClone) supplemented with 10% fetal bovine serum (FBS) (Gibco) and 1% antibiotic/antimycotic (Gibco). Cells were maintained in a humidified 5% CO2 incubator at 37°C. Antibodies used for the immunoblot and immunoprecipitation analysis are as follows, anti-Flag (Cell Signaling, 8146), anti-Strep (Qiagen, 34850), anti-GST (Santa Cruz, sc-138), anti-IRF3 (Abcam, ab25950), anti-phospho IRF3 (Ser396) (Cell Signaling, 4947), anti-p65 (Cell Signaling, 4764S), anti-phospho p65 (Cell Signaling, 3031S), anti-TBK1 (Cell Signaling, 3504S), anti-phospho-TBK1 (Cell Signaling, 5483S), anti-β-actin (Santa Cruz,SC 47778), RIG-I (D14G6; 3743), MDA-5 (D74E4; 5321), Anti-FMDV 2B (homemade), anti-Caspase8 (Cell Signaling, 9746S), anti-Caspase3 (Cell Signaling, 9662S) and anti-β-actin (Santa Cruz,SC 47778)

### Preparation of stable cell lines

To prepare FMDV 2B overexpressing stable cell line, RAW264.7 cells were seeded in 6-well culture plates 12 hours after seeding (60% cell confluence), pIRES-Flag-2B plasmid was transfected into the cells by incubating with Lipofectamine 2000 (Invitrogen) and for 6 hours. Next, culture media was changed into complete DMEM and incubated for 12 hours before transferring the cells into a 100 mm culture dish. Cells were incubated with complete DMEM media, and after cells were attached, media was replaced with complete DMEM with 2 μg/ml puromycin. Replace media with fresh puromycin containing 10% DMEM every 2 days until resistant colonies appear. Expression of Flag-tagged 2B was confirmed by immunoblotting.

### Virus propagation, infection, and stimulant transfection

Recombinant Green Fluorescent Protein (GFP) expressing viruses such as H1N1 influenza A virus (A/PR8/8/34; PR8-GFP) and Newcastle disease virus (NDV-GFP) were propagated in allantoic fluid of 10-day old embryonated chicken eggs. And GFP-expressing vesicular stomatitis virus (VSV-GFP), GFP-expressing coxsackievirus (H3-GFP), and EV-71 were propagated in the monolayer of *Ceropithecus aethiops* epithelial kidney (Vero; ATCC^®^ CCL-81™) cells, and virus titer was determined by the plaque assay. During virus infection, we changed the culture media to 1% FBS containing DMEM, and viruses were added to the medium with the indicated multiplicity of infection (MOI). After 2 hours of infection, the media was changed to complete DMEM. Poly (I·C) was transfected with Lipofectamine 2000 into HEK293T cells or used to treat RAW264.7 cells. 5’- triphosphate double-stranded RNA (5’ppp-dsRNA, Invivogen) was transfected into both cell lines with Lipofectamine RNAiMAX (Invitrogen) to stimulate immune response pathways. According to the manufacturer’s instructions, cell lysates were harvested at the indicated time, and GFP expression was measured with the Glomax multi-detection system (Promega, Wisconsin, USA).

### Virus infection and stimulant transfection

GFP-expressing H1N1 influenza A virus (A/PR8/8/34; PR8-GFP) and GFP-expressing Newcastle disease virus (NDV-GFP) were propagated in the allantoic fluid of 10-day-old specific-pathogen-free embryonated chicken eggs GFP-expressing vesicular stomatitis virus (VSV-GFP), GFP-expressing Coxsackievirus (H3-GFP), and EV-71 were propagated on a monolayer of Vero cells. Cultured cells medium was replaced with DMEM or RPMI containing 1% FBS before virus infection, and the viruses were added into the medium with the indicated MOI. After 2 h of incubation, the extracellular virus was removed and replaced with 10% FBS DMEM or RPMI. Poly(I:C) (Invivogen) was transfected to HEK293T cells with Lipofectamine 2000 (Invitrogen) and treated to RAW264.7 cells, respectively. 5’ppp-dsRNA (Invivogen, tlrl-3prna) was transfected to both cell lines with Lipofectamine RNA iMAX (Invitrogen) to stimulate immune response pathways. According to the manufacturer’s instructions, cell lysates were harvested at the indicated time, and GFP expression was measured with the Glomax multi-detection system (Promega, Wisconsin, USA).

### FMDV infection into cell culture

Fetal porcine kidney (LFBK) cells were used to perform the FMDV infection experiment. The cell monolayer was prepared in cell culture plates (90x20mm) and incubated overnight, followed by infection with FMDV Asia1/Shamir at 0.1MOI for 2 hours before replacing with complete DMEM culture media. For the RIG-I ubiquitination assay, cells were treated with MG132 (10µM) for 6 hours before collecting the cells. Cells were collected at indicated time points after infection, RIG-I and MDA5 expression levels, RIG-I ubiquitination and RIG-I interaction with RNF125 and FMDV 2B were examined by immunoblotting.

### Determination of virus titer

To evaluate the virus titer of cell culture supernatants, plaque assays or TCID50 was performed. Cell culture supernatants of growing cells or freeze-thawed cells were used to titrate VSV-GFP, H3-GFP, or PR8-GFP, respectively. The supernatants were serially 10-fold diluted and inoculated into Vero cells in 1% FBS containing DMEM. Following 2 h incubation at 37°C, the inoculums were removed and replaced with DMEM containing 0.1% agarose (Sigma-Aldrich). Plates were then incubated for another 36 hr at 37°C and examined for plaque formation at 200× magnification. The virus titer was calculated using the number of plaque-forming units and the dilution factor. TCID50 was carried out for EV-71 virus titration. Briefly, 100 µL of Vero cells cultured were prepared at 96 well flat-bottomed cell culture plates, and 100 µL of 10-fold serial dilutions of virus suspensions were added to plates, with each dilution being repeated in eight wells and incubated at 37°C for 3-4 days. CPE was observed in each well under a light microscope, and TCID50 was calculated using the method described by Reed and Muench (65).

### mRNA expression analysis by qRT-PCR

FMDV 2B or pIRES empty vector stably expressing RAW264.7 cells or HEK293T cells were infected with VSV-GFP or PR8-GFP virus, and cells were collected in different time courses. Total mRNA was obtained from cells using RNeasy Mini Kit (Qiagen), and cDNA synthesis was performed using a ReverTra Ace kit (Toyobo). qRT-PCR analysis was performed using QuantiTect SYBR Green PCR kit (Qiagen) on a Rotor-Gene Q (Qiagen). The target gene expression was normalized to the expression of the glyceraldehyde-3-phosphate dehydrogenase (GAPDH) reference gene. The primer sequences for genes used for real-time PCR are listed in **Table 1**.

**Table 1:** The primer sequences for the genes used in real-time PCR.

Target gene	Forward primer	Reverse primer
IFN-β	TCCAAGAAAGGACGAACATTCG	TGCGGACATCTCCCACGTCAA
IL-6	GACAACTTTGGCATTGTGG	ATGCAGGGATGATGTTCTG
ISG-15	CAATGGCCTGGGACCTAAA	CTTCTTCAGTTCTGACACCGTCAT
ISG-56	AGAGAACAGCTACCACCTTT	TGGACCTGCTCTGAGATTCT
GBP1	AAAAACTTCGGGGACAGCTT	CTGAGTCACCTCATAAGCCAAA
IFN-α	ATAACCTCAGGAACAACAG	TCATTGCAGAATGAGTCTAGGAG
Mx1	ACAAGCACAGGAAACCGTATCAG	AGGCAGTTTGGACCATCTTAGTG
OAS-1β	AGGTGGTAAAGGGTGGCT	TGCTTGACTAGGCGGATG
TNF-α	AGCAAACCACCAAGTGGAGGA	GCTGGCACCACTAGTTGGTGGT
GAPDH Mouse	AGCAAACCACCAAGTGGAGGA	GACGGACACATTGGGGGTAG

### Immunoprecipitation

Cells were co-transfected with indicated plasmids, and 36 h post-transfection cells were harvested. Whole-cell lysates (WCL) were obtained after lysis with protease inhibitor cocktail- and phosphatase inhibitor cocktail (Sigma)-containing radioimmunoprecipitation assay (RIPA) lysis buffer (50 mM Tris-HCl, 150 mM NaCl, 0.5% sodium deoxycholate, 1% IGEPAL, 1 mM NaF, 1 mM Na3VO4) and sonication with a sonicator (Sonics). The WCLs were pre-cleared with Sepharose 6B (GE Life Sciences) at 4°C for 2 h. The pre-cleared whole cell lysates were incubated overnight with 2 μg target protein antibodies, 50% slurry of glutathione-conjugated Sepharose (GST) beads (Amersham Biosciences) or Strep beads (IBA Life Sciences) with agitation at 4°C. Only for the whole cell lysate incubated with antibodies, 20 μl protein, A/G PLUS-agarose was added to precipitate proteins attached with antibodies. The immunoprecipitated beads were collected after centrifugation and were washed with lysis buffer under different washing conditions. Precipitates were subjected to SDS-PAGE, and immunoblotting was performed with the appropriate antibody.

### Immunoblot analysis

Cell lysates or immunoprecipitated beads were mixed with 2× sample buffer (Sigma), samples were loaded onto SDS-PAGE, and proteins were separated by molecular weight. Samples were separated by SDS-PAGE and transferred onto a PVDF membrane (Bio-rad) in a buffer containing 30 mM Tris, 200 mM glycine, and 20% methanol for 2 hr. The membranes were blocked with Tris-buffered saline containing 0.05% Tween 20 (TBST) and 5% bovine serum albumin (BSA) for 1 h and incubated with target antibodies overnight at 4°C. To detect target proteins, the membranes were washed with phosphate-buffered saline containing 0.05% Tween 20 (PBST) or TBST and incubated with a 1:3,000 dilution of horseradish peroxidase (HRP)-conjugated secondary antibodies for 1 h, and the HRP on the membranes were developed with Western blotting detection reagents (GE Healthcare; ECL Select Western blotting detection reagent) and visualized using an ImageQuant LAS 4000 (GE Life Sciences).

### Luciferase reporter assay

HEK293T cells were transfected with 400ng of firefly luciferase reporter plasmid (pGL-3), 100ng of pRL-TK (Renilla luciferase- as an internal control) (Promega), increasing amounts of FMDV 2B-Flag (0-1600ng) expression plasmid and pIRES empty vector to equalize the transfection amounts using Polyethylenimine (PEI) transfection reagent. To stimulate the IFN-β promoter, plasmids carrying the RIG-I, MAVS, TRIF, and TBK1 gene were co-transfected together with luciferase reporter plasmids or PR8-GFP, NDV-GFP viruses infection, poly(I·C) transfection were conducted at 12 h post-transfection. At 12 h after stimulation, the cells were washed with PBS and lysed with 1X Passive Lysis buffer (Promega) for 20 minutes. Following the manufacturer’s instruction, luciferase activity was measured using the Dual-Luciferase Reporter Assay System (Promega; E1980). Luciferase activity in cells expressing only IFN-β reporter and Renilla plasmids was measured as a control. Data are expressed in accordance with relative firefly luciferase activity normalized against Renilla luciferase activities.

### Immunofluorescence and confocal analysis

The HeLa cells were seeded in collagen-coated chamber slides (LabTek, Nunc). 12 hours later, cells were transfected with the indicated plasmids and incubated for 36 hours (MG132 treatment was done 28 hours after transfection). After 36 hours of incubation, the cultured cells were washed with phosphate buffer saline (PBS) and fixed with 4% paraformaldehyde for 20 min, then permeabilized through incubation for 20 min with 100% methanol at -20°C. The fixed cells were first incubated with 2% FBS diluted in PBS for 1 hour to block the non-specific binding of antibodies. Next, cells were incubated with indicated antibodies for 12 hours in 4°C.

After three times washing with PBS, cells were incubated with the 1:100 diluted secondary antibodies for 1 hr at room temperature. After three times PBS washing, the nuclei were visualized following incubation for 10 min with 1:50000 diluted DAPI (Sigma) adding 1 mg/ml RNase A, and the slides were mounted with the mounting solution (VECTOR). Images were captured using a Nikon C2 Plus confocal microscope (Nikon), consisting of a Nikon Eclipse Ti inverted microscope with a confocal scanning system (Nikon) in conjunction with a C-HGFIE precentered fiber illuminator (Nikon). Fluorescein isothiocyanate (FITC) and tetramethylrhodamine isocyanate (TRITC) fluorescence were detected using the 488-nm and 561-nm laser lines of a Sapphire driver unit (Coherent), respectively, and DAPI fluorescence was detected using the 405-nm laser line of a Cube laser system (Coherent). The image data were analyzed using NIS Elements microscope imaging software (Nikon).

### Cell apoptosis assay

HEK293T and PK-15 cells were seeded in 6 well plates and incubated for 12 hours. After incubation, for 12 hours, cells were transfected with indicated plasmids. 12 hours after transfection, cells were removed and seeded in 96 well plates. Cell apoptosis and proliferation were measured in given time points using RealTime-Glo™ Annexin V Apoptosis and Necrosis Assay (Promega, JA1000), CellTiter 96^®^ Non-Radioactive Cell Proliferation Assay-MTT (Promega, G4000), and Cell Counting Kit-8 (Dojindo Molecular Technologies) according to manufacturer’s instructions.

### RNA interference

For RNF125 knockdown, we transfected HEK293T cells with 50 nM of RNF125-specific siRNA oligos or non-targeting control siRNA (Bioneer, Korea) using Lipofectamine 2000 (Invitrogen) for 48 h. The sequence targeting human RNF125 was: 5’-CCGUGUGCCUUGAGGUGUU-3’.

### Statistical analysis

Data are presented as the means and standard deviations (SD) and represent at least two independent experiments. Statistical analysis was performed with Student’s t-test in GraphPad Prism 6 software. P values are indicated in the legends. *P < 0.05, **P < 0.01, ***P < 0.001, ****P < 0.0001.

## Data availability statement

The original contributions presented in the study are included in the article/**Supplementary Material**. Further inquiries can be directed to the corresponding authors.

## Author contributions

AW and MBU performed most of the experiments. J-HC, PP, SS, and KC helped with the experiments and contributed to the discussions. AW, MBU, J-HP, and J-SL designed the study. AW, MBU, and J-SL wrote the manuscript. J-HP and J-SL supervised the study. All the authors helped with data analysis. All authors contributed to the article and approved the submitted version.

## Funding

This work was supported by the National Research Foundation (Grant No. 2018M3A9H4079660, 2019R1A2C2008283, 2021R1A6A1A03045495) and Korea Research Institute of Bioscience and Biotechnology Research Initiative Program (KGM9942011), Republic of Korea.

## Conflict of interest

The authors declare that the research was conducted in the absence of any commercial or financial relationships that could be construed as a potential conflict of interest.

## Correction note

A correction has been made to this article. Details can be found at: 10.3389/fimmu.2026.1959310.

## Publisher’s note

All claims expressed in this article are solely those of the authors and do not necessarily represent those of their affiliated organizations, or those of the publisher, the editors and the reviewers. Any product that may be evaluated in this article, or claim that may be made by its manufacturer, is not guaranteed or endorsed by the publisher.
