# Correction: Foot-and-mouth disease virus non-structural protein 2B downregulates the RLR signaling pathway via degradation of RIG-I and MDA5

**DOI:** 10.3389/fimmu.2026.1959310

**Published:** 2026-09-10

**Authors:** Asela Weerawardhana, Md Bashir Uddin, Joo-Hyung Choi, Prabuddha Pathinayake, Sung Ho Shin, Kiramage Chathuranga, Jong-Hyeon Park, Jong-Soo Lee

**Affiliations:** 1 College of Veterinary Medicine, Chungnam National University, Daejeon, Republic of Korea; 2 Department of Medicine, Sylhet Agricultural University, Sylhet, Bangladesh; 3 Department of Microbiology and Immunology, University of Texas Medical Branch, Galveston, TX, United States; 4 Foot and Mouth Disease Division, Animal Quarantine and Inspection Agency, Anyang, Republic of Korea; 5 Wildlife Disease Response Team, National Institute of Wildlife Disease Control and Prevention (NIWDC), Gwangju, Republic of Korea; 6 Immune Health Program, Hunter Medical Research Institute, University of Newcastle, Newcastle, NSW, Australia

**Keywords:** foot and mouth disease virus (FMDV), 2B, RIG-I, MDA5, RNF125

There was a mistake in **Figure 1** as published. The cell images presented in **Figure 1A** and **1E** were originally generated as part of a broader screening study investigating the roles of FMDV proteins in host immune regulation. These experiments included multiple biological and technical replicates. During the final preparation of figures for related manuscripts, the control vector phenotype images were inadvertently cross-referenced, resulting in the reuse of the same control images. During our archive review, we identified alternative control images from the original experiments that had not been used in any other manuscript. These images represent only the control conditions, and their replacement does not affect the experimental results, data interpretation, or the conclusions of the study.

**Figure 1:**
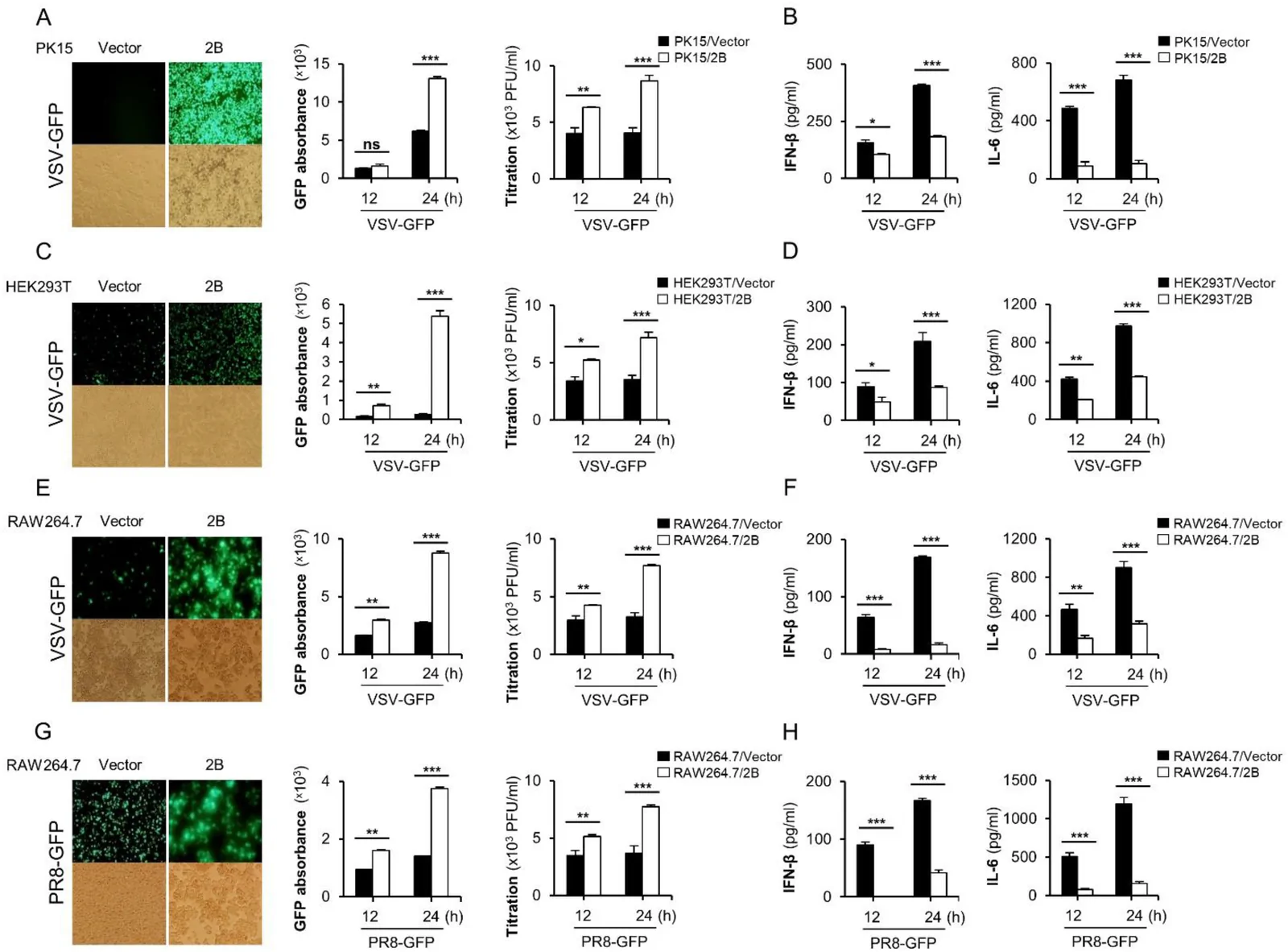
FMDV 2B negatively regulate RNA virus-mediated innate immune responses. PK-15 cells **(A, B)** and HEK293T cells **(C, D)** were transiently transfected with the control vector or FMDV 2B and infected with VSV-GFP. GFP expression, GFP absorbance, and virus titer was taken at 12 and 24 hpi **(A, C)**. The concentration of secreted IFN-b and IL-6 in supernatants was determined at 12 and 24 hpi by ELISA **(B, D)**. Control vector and FMDV 2B stably expressing RAW264.7 cells were infected with VSV-GFP **(E, F)** and PR8-GFP **(G, H)**. GFP expression, GFP absorbance, and virus titer was taken at 12 and 24 hpi **(E, G)**. The concentration of IFN-b and IL-6 secreted in supernatants were determined at 12 and 24 hpi by ELISA **(F, H)**. Results representative of at least two independent experiments, each with similar results, and the values are expressed as mean ± SD of three biological replicates. Student’s t-test; *p < 0.05; **p < 0.01; ***p < 0.001; ns, not significant.

The corrected **Figure 1** appears below.

The original version of this article has been updated.

