# Supplementary material for: Foot-and-mouth disease virus non-structural protein 2B downregulates the RLR signaling pathway via degradation of RIG-I and MDA5
Source: Front Immunol. 2022 Sep 29;13:1020262. doi: 10.3389/fimmu.2022.1020262 (PMC9556895; doi:10.3389/fimmu.2022.1020262)
Supplement: Supplementary file 1 [file DataSheet_1.pdf]

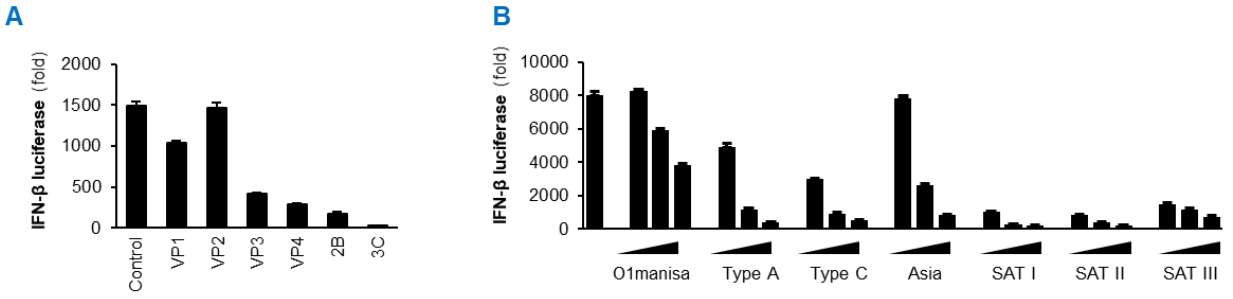

Supplementary Figure 1. FMDV 2B-mediated negative regulation of type-I IFN signaling is consistent with all FMDV serotypes. (A and B) HEK293T cells were transfected with interferon-β promoter encoding firefly luciferase plasmid, TK-Renilla plasmid, (A) plasmids containing different genes of FMDV genome, or (B) increasing doses of FMDV 2B gene-containing plasmids from 7 FMDV serotypes (O1manisa, Type A, Type C, Asia, SAT I, SAT II, SAT III) together with RIG-I 2CARD expressing plasmid for 24 hours. Results are expressed relative to those of Renilla luciferase alone (internal control). Results are representative of at least two independent experiments, each with similar results, and the values are expressed as mean ± SD of three biological replicates.

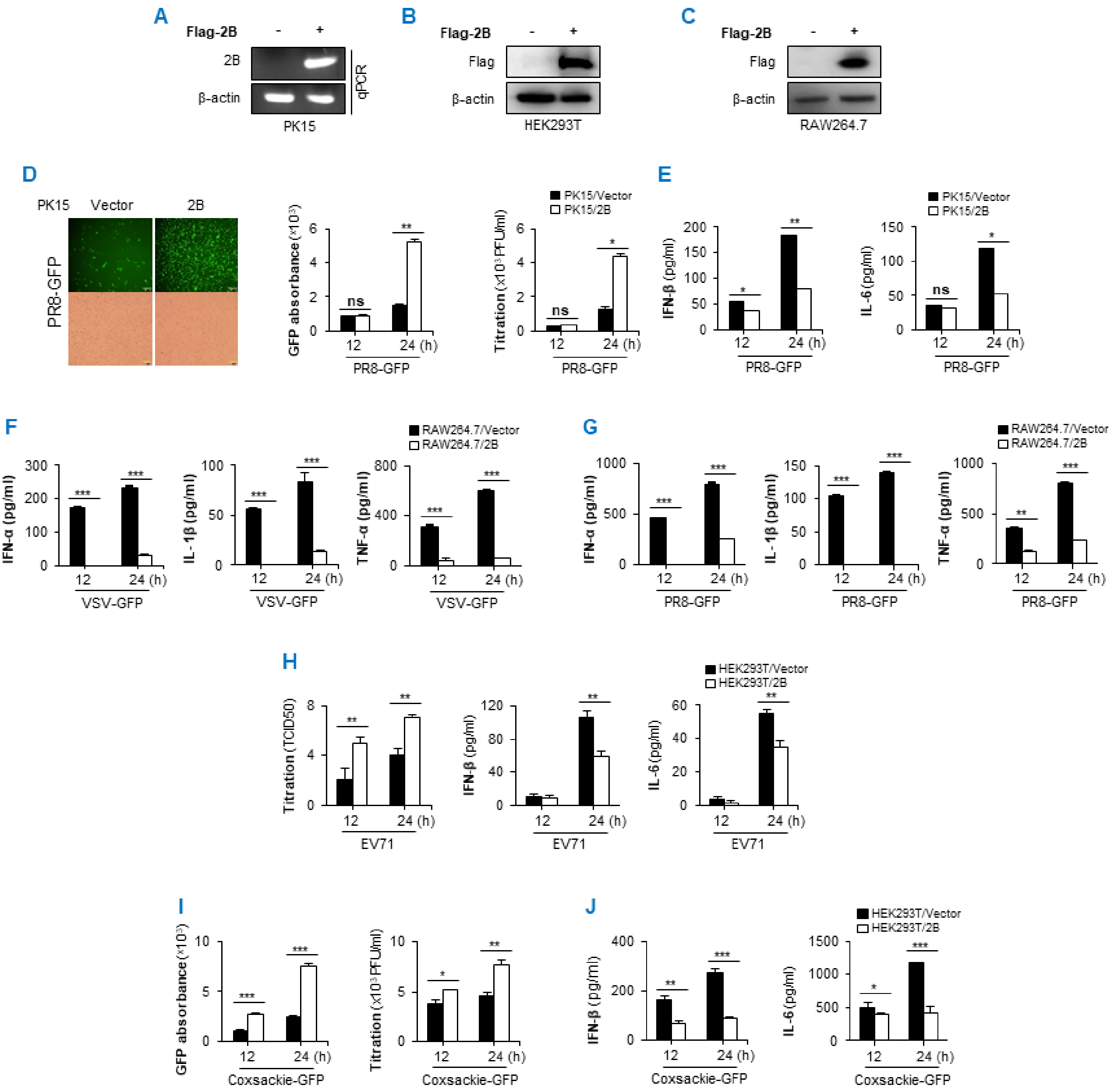

Supplementary Figure 2. FMDV 2B suppresses RNA virus-mediated innate immune response and enhances viral replication. PK15 cells (A) HEK293T cells (B) were transfected with Flag-2B plasmid or control vector and mRNA expression was determined by qPCR (A) or whole cell lysates were immunoblotted with indicated antibodies (B). Control vector or FMDV 2B stably overexpressing cell lysates were immunoblotted with indicated antibodies (C). PK-15 cells were transiently transfected with the control vector or FMDV 2B and infected with PR8-GFP. GFP expression, GFP absorbance and virus titer was taken at 12 and 24 hpi (D). The concentration of secreted IFN-β and IL-6 in supernatants was determined at 12 and 24 hpi by ELISA (E). Control vector and FMDV 2B stably expressing RAW264.7 cells were infected with VSV-GFP (F) and PR8-GFP (G). The concentration of IFN-α, IL- 1β, and TNF-α secreted in supernatants were determined at 12 and 24 hpi by ELISA.

HEK293T cells were transiently transfected with FMDV 2B plasmids and infected with EV71, Virus titration was measured by TCID50 assay, and the concentration of IFN-β and IL-6 secreted in supernatants were determined at 12 and 24 hpi by ELISA (H). HEK293T cells were transiently transfected with FMDV 2B plasmids and infected with Coxsackie virus-GFP. Viral replication was measured by fluorescence microscopy, GFP absorbance, and plaque assay at indicated time points (I). The concentration of secreted IFN-β and IL-6 in supernatants was determined at 12 and 24 hpi by ELISA (J). Results representative of at least two independent experiments, each with similar results, and the values are expressed as mean ± SD of three biological replicates. Student's t-test; * $p < 0.05$; ** $p < 0.01$; *** $p < 0.001$, ns; not significant.

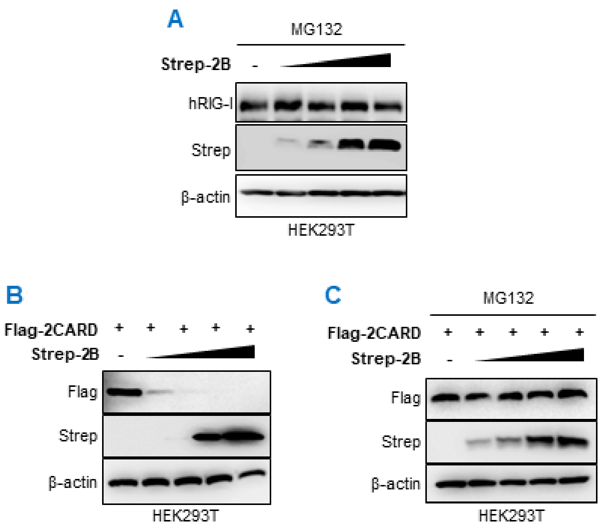

Supplementary Figure 3. FMDV 2B mediates proteosomal degradation of RIG-I. HEK293T cells were transfected with increasing doses of Strep-FMDV 2B plasmids and treated with MG132 for 6 hours before harvesting the cells. Whole-cell lysates were immunoblotted with indicated antibodies (A). HEK293T cells were transfected with Flag-RIG-I 2CARD together with increasing doses of Strep-FMDV 2B plasmids and not treated (B) or treated (C) with MG132 for 6 hours before harvesting the cells. Whole-cell lysates were immunoblotted with indicated antibodies. Results representative of at least two independent experiments, each with similar results.

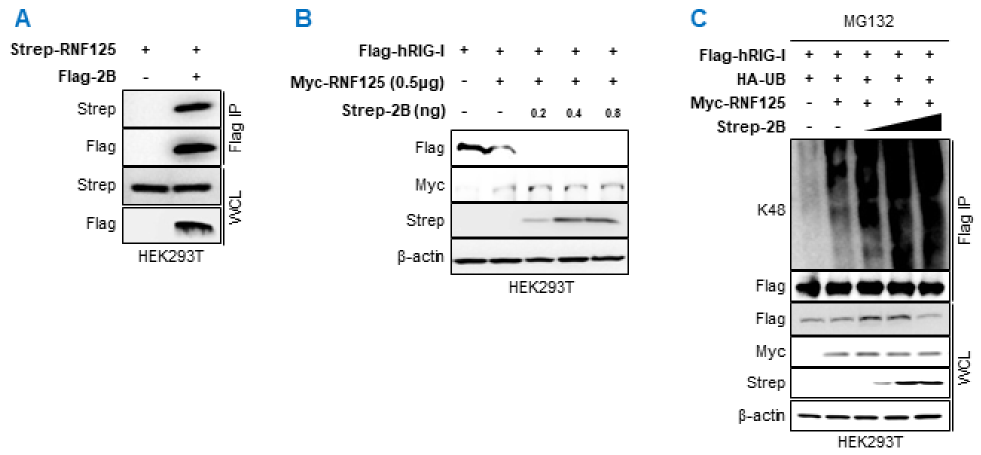

Supplementary Figure 4. RNF125 is essential for FMDV 2B-mediated K48-linked polyubiquitination and degradation of RIG-I. HEK293T cells were transfected with Strep-RNF125 together with Flag-2B or control vector containing plasmids. Whole-cell lysates were subjected to flag immunoprecipitation and immunoblotted with indicated antibodies (A). HEK293T cells were transfected with Flag-RIG-I, Myc-RNF125 together with increasing doses of Strep-FMDV 2B plasmids. Whole-cell lysates were immunoblotted with indicated antibodies (B). HEK293T cells were transfected with Flag-RIG-I, HA-ubiquitin, Myc-RNF125 together with increasing doses of Strep-2B plasmids and treated with MG132 for 6 hours before harvesting the cells. Whole-cell lysates were subjected to Flag immunoprecipitation and immunoblotted with indicated antibodies (C). Results representative of at least two independent experiments, each with similar results.

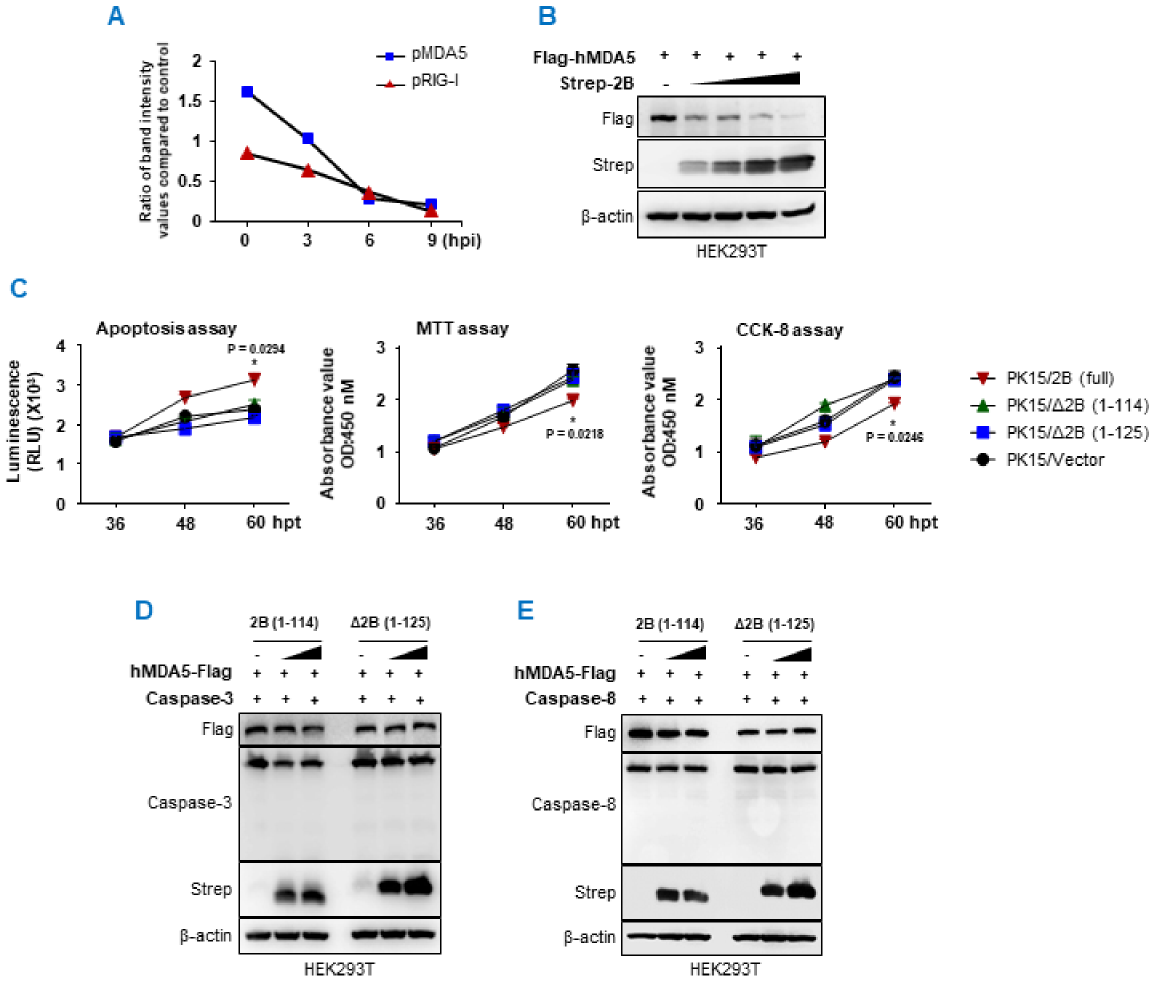

Supplementary Figure 5. The C-terminal region of FMDV 2B is essential for apoptosis and degradation of MDA5. Band intensity of figure 6A was quantified using ImageJ and data are represented as fold-change compared to control (A). HEK293T cells were transfected with Flag-MDA5 together with increasing doses of Strep-FMDV 2B plasmids. Whole-cell lysates were subjected to immunoblotting with indicated antibodies (B). PK-15 cells were transfected with Strep-FMDV 2B wild-type, FMDV Δ2B (1-114) mutant, FMDV Δ2B (1-125) mutant, and control vector. Annexin V apoptosis assay, CCK-8 assay, and MTT assay were conducted at indicated time points post-transfection (C). (D and E) HEK293T cells were transfected with Flag-MDA5, caspase-3 (D), or caspase-8 (E) together with increasing doses of Strep-FMDV Δ2B (1-114) or FMDV Δ2B (1-125) plasmids. Whole-cell lysates were immunoblotted with indicated antibodies. Results representative of at least two independent experiments, each with similar results, and the values are expressed as mean ± SD of three biological replicates. Student’s t-test; * $p < 0.05$.
